# Unveiling the key genes, environmental toxins, and drug exposures in modulating the severity of ulcerative colitis: a comprehensive analysis

**DOI:** 10.3389/fimmu.2023.1162458

**Published:** 2023-07-19

**Authors:** Yao Wang, Hao Zhuang, Xiao-han Jiang, Rui-han Zou, Hai-yang Wang, Zhi-ning Fan

**Affiliations:** Digestive Endoscopy Department, Jiangsu Province Hospital, The First Affiliated Hospital with Nanjing Medical University, Nanjing, China

**Keywords:** ulcerative colitis, microarray, biomarker, genomics, bioinformatics

## Abstract

**Background:**

As yet, the genetic abnormalities involved in the exacerbation of Ulcerative colitis (UC) have not been adequately explored based on bioinformatic methods.

**Materials and methods:**

The gene microarray data and clinical information were downloaded from Gene Expression Omnibus (GEO) repository. The scale-free gene co-expression networks were constructed by R package “WGCNA”. Gene enrichment analysis was performed *via* Metascape database. Differential expression analysis was performed using “Limma” R package. The “randomForest” packages in R was used to construct the random forest model. Unsupervised clustering analysis performed by “ConsensusClusterPlus”R package was utilized to identify different subtypes of UC patients. Heat map was established using the R package “pheatmap”. Diagnostic parameter capability was evaluated by ROC curve. The”XSum”packages in R was used to screen out small-molecule drugs for the exacerbation of UC based on cMap database. Molecular docking was performed with Schrodinger molecular docking software.

**Results:**

Via WGCNA, a total 77 high Mayo score-associated genes specific in UC were identified. Subsequently, the 9 gene signatures of the exacerbation of UC was screened out by random forest algorithm and Limma analysis, including BGN,CHST15,CYYR1,GPR137B,GPR4,ITGA5,LILRB1,SLFN11 and ST3GAL2. The ROC curve suggested good predictive performance of the signatures for exacerbation of UC in both the training set and the validation set. We generated a novel genotyping scheme based on the 9 signatures. The percentage of patients achieved remission after 4 weeks intravenous corticosteroids (CS-IV) treatment was higher in cluster C1 than that in cluster C2 (54% *vs*. 27%, Chi-square test, *p*=0.02). Energy metabolism-associated signaling pathways were significantly up-regulated in cluster C1, including the oxidative phosphorylation, pentose and glucuronate interconversions and citrate cycle TCA cycle pathways. The cluster C2 had a significant higher level of CD4+ T cells. The”XSum”algorithm revealed that Exisulind has a therapeutic potential for UC. Exisulind showed a good binding affinity for GPR4, ST3GAL2 and LILRB1 protein with the docking glide scores of –7.400 kcal/mol, –7.191 kcal/mol and –6.721 kcal/mol, respectively.We also provided a comprehensive review of the environmental toxins and drug exposures that potentially impact the progression of UC.

**Conclusion:**

Using WGCNA and random forest algorithm, we identified 9 gene signatures of the exacerbation of UC. A novel genotyping scheme was constructed to predict the severity of UC and screen UC patients suitable for CS-IV treatment. Subsequently, we identified a small molecule drug (Exisulind) with potential therapeutic effects for UC. Thus, our study provided new ideas and materials for the personalized clinical treatment plans for patients with UC.

## Introduction

As a chronic relapsing bowel disease, Ulcerative colitis (UC) is characterized by intestinal inflammation, mucosal injury, and fibrosis (1). The most common symptoms of UC are bloody diarrhoea, weight loss and abdominal pain. UC has represented an increasing prevalence worldwide and carried a significant global disease burden in the past few years (2). Aggravating and relieving factors of UC remains undefined, yet multiple genetic and environmental factors have been demonstrated to participate in its severity and progression (3–6). With the rapid development of high-throughput sequencing, bioinformatic analysis of gene expression profiling has been widely applied to investigate molecular mechanisms and identify potential therapeutic targets (7–9). However, few studies have explored the underlying mechanisms and biomarkers of exacerbation and remission for UC based on bioinformatic methods.

In many high-quality studies, the severity of disease was scored using the Mayo score for UC (10–13). The Mayo score ranges from 0 to 12, with higher scores indicating more severe disease (14). The Mayo score consists of four items: stool frequency, rectal bleeding, findings of flexible proctosigmoidoscopy and the clinical assessment (15). Mayo score can also be used to assess the disease activity and efficacy of the therapeutic regimen for UC (13).

In this study, we aim to investigate the key gene alterations affecting the severity of UC based on Mayo score and bioinformatic analysis, contributing to the development of personalized clinical management and treatment regimens for UC. The workflow chart of our study was shown in **Supplementary Figure 1**.

## Materials and methods

### Data acquisition

The microarray data and clinical information of UC patients were downloaded from the Gene Expression Omnibus (GEO) (GSE109142, and GSE92415) (16). The GSE109142, and GSE92415 cohorts contained Mayo scores information for all samples. Sample sizes: GSE109142 (Normal, n = 20; UC, n = 206); GSE92415 (Normal, n = 21; UC, n = 162). The microarray data was download at https://www.ncbi.nlm.nih.gov/geo/ on December 1, 2022. The GSE109142 cohort was used as the training set considering its relatively large sample size. The GSE92415 cohort was used as the validation set. Furthermore, another independent validation dataset (GSE73661) was then obtained from GEO database, in which 166 UC patients for whom mayo endoscopic score were available.

### Weighted correlation network analysis

R package “WGCNA” was utilized to construct the co-expression networks based on the microarray data (17). As a soft-thresholding power, the primary role of β was to emphasize strong correlations between the genes and penalize weak correlations. The topological overlap matrix (TOM) was transformed from the adjacency after we chosed the β based on the “pickSoftThreshold” algorithm which came with the “WGCNA” R package (18). Pearson’s correlation analysis was conducted to appraise the correlation between module eigengenes (MEs) and Mayo score. Subsequently, gene module with the highest pearson’s coefficient was considered as the module most relevant to the Mayo score (Mayo score-related module) in UC. We set the screening criteria as |MM| > 0.8 and |GS| > 0.1, and then we obtained the distinct hub genes in the Mayo score-related module (9). Specific schematic process of WGCNA can be found in **Supplementary Figure 2**.

WGCNA was performed separately on GSE109142 and GSE92415 to determine Mayo score-related hub genes, respectively. The intersection of the hub genes form GSE109142 and GSE92415 was included in the next step of analysis and the results was visualized using the “VENNY 2.1” online tool (19) (https://bioinfogp.cnb.csic.es/tools/venny/index.html).

### Gene enrichment analysis

The Metascape database was utilized to perform enrichment analyses (20). All other parameters set as default. Terms with a *p value* < 0.01, minimum count of 3, and an enrichment factor > 1.5 were utilized in the next step of the analysis. Using screening criteria of kappa scores = 4 and similarity > 0.3, Metascape was utilized to perform hierarchical clustering to partition enrichment terms into distinct clusters, and the terms with the minimum *p value* were selected as the representative terms.

Gene Set Enrichment Analysis (GSEA) software (version 3.0) (http://software.broadinstitute.org/gsea/index.jsp) was used to perform GSEA analysis and identify significantly enriched pathways in different group (21). In the GSEA runs, maximum gene set size was set to 5,000 and minimum gene set size was set to 5. FDR ≤ 0.25 were considered as statistically significant. The KEGG pathways (c2.cp.kegg.v7.4.symbols.gmt) were arranged according to the normalized enrichment scores (NES) (22). Top five significantly enriched KEGG pathways were shown.

### Linear models for microarray data (Limma) analysis and random forest

Based on upper and lower quartiles of the set of Mayo scores in training set, UC patients were stratified to low-, moderate-, and high-Mayo score groups. Substantially, Limma analysis and random forest was used to screen the key gene signatures from the intersection of the hub genes (23). Differential gene expression analysis followed the Limma pipeline performed by R package “limma” (version 3.40.6). Differential expression genes (DEGs) were identified according to the filter criteria (|fold change| > 1.5, FDR < 0.05). The random forest algorithms was performed by the “randomForest” packages in R (24). The ‘randomForest’ package in R was used to grow a forest of 500 trees using the default settings. Based on the “randomForest” algorithms, we selected the top 10 genes with the highest importance for downstream analysis. The intersection of the results between Limma and random forest methods was identified as the key gene signatures.

### Unsupervised hierarchical clustering

Unsupervised clustering was performed through R package “ConsensusClusterPlus”, using agglomerative pam clustering with a 1-pearson correlation distances and resampling 80% of the samples for 10 repetitions (25). The optimal number of clusters was determined using the empirical cumulative distribution function plot. We divided UC patients into different molecular patterns based on the expression matrix of key gene signatures obtain by Limma and random forest methods.

### Identification of immune infiltration characterization of UC

The Immune Cell Abundance Identifier (ImmuCellAI) database was used to estimate the abundance of 24 types of immune cells in GSE109142 by inputting microarray data (26). ImmuCellAI database is a online tool to estimate the abundance of the 24 immune cells, comprising of 18 T-cell subtypes and 6 other immune cells: B cell, NK cell, Monocyte cell, Macrophage cell, Neutrophil cell and DC cell.

### Discovery of potential drugs by computational methods

A similarity scoring algorithm called eXtreme Sum (XSum) was performed to screen the candidate small molecule drugs based on the connectivity map (cMap) database (27). The DEGs between different immune infiltration subtypes were used as input file of “XSum” algorithm. Subsequently, a score was calculated for each small molecule drugs of cMap database by “XSum” algorithm. Lower score indicates greater potential to act as a therapeutic drug for reversing the immune infiltration condition.

RCSB Protein Data Bank (PDB) (www.rcsb.org/pdb/home/home.do) was used to obtain the crystal structures of proteins coded by the hub gene (28). Furthermore, the 3D structure of the small molecule drugs was download from PubChem (https://www.ncbi.nlm.nih.gov/pccompound) (29). The molecular docking process involved preparing the proteins and ligands, setting up a grid, and docking the compounds; these were conducted using the Schrodinger software (30). The best pose was choose based on the docking score and the rationality of molecular conformation.

### Chemical-gene interaction analysis

To explore the interplay between environmental chemical toxicant exposure and the UC exacerbation, we conducted an analysis utilizing the meticulously curated research studies on the Comparative Toxicogenomic Database (CTD). In our analysis, we scrutinized environmental toxicants and drugs affecting the gene expression of all key genes previously identified. Our analysis is limited to human species only.

### Real time quantitative PCR detection of GPR4, ST3GAL2, and LILRBgene expression

TRIzol reagent (Ambion, USA) was utilized for total RNA extraction, followed by reverse transcription of the extracted mRNA into cDNA using PrimeScript™ RT Master Mix (Takara, Japan). RT-qPCR was performed to quantify the transcripts using ChamQ SYBR qPCR Master Mix (Vazyme, China). Through RT-qPCR, gene expression was detected and the relative expression levels of the genes were evaluated using the 2-ΔΔCT method. To serve as an internal reference, GAPDH was used and the experiment was repeated thrice to establish the average. The following primer sequences were utilized for the detection of GPR4, ST3GAL2, and LILRB1 expression levels:

The forward primer of GPR4 was 5’-CATCGTGCTGGTCTGCTT-3’.
The reverse primer of GPR4 was 5’-CACAGTTGAGGCTGGTGAA-3’.
The forward primer of ST3GAL2 was 5’-TTCACCTACTCGCACCACA-3’.
The reverse primer of ST3GAL2 was 5’-CGACAGGCACAGCTCTTG-3’.
The forward primer of LILRB1 was 5’-CCTTGTGGGCACTCCATT-3’.
The reverse primer of LILRB1 was 5’-ACGCATCTCGCATCTGG-3’.

Four patients with UC and four healthy controls who have signed informed consents were recruited from Jiangsu Provincial People’s Hospital. Samples of inflamed intestinal tissue from UC patients and normal tissue were harvested from colonoscopy biopsy specimens of both patients and controls.

### Statistical analyses

R software (version 4.0.4) was utilized for all statistical procedures. Continuous variables were compared with the Wilcoxon/Kruskal–Wallis test. Differences in proportion were tested by the chi-square test. A *p value* less than 0.05 was considered significant. Receiver operating characteristic (ROC) curve was constructed to assess the predictive efficacy (31). Dimensionality reduction was performed using principal component analysis (PCA), uniform Manifold Approximation and Projection (UMAP) t-distributed stochastic neighbor embedding (tSNE) (32–34).

## Results

### Mayo score-related gene module revealed by WGCNA

**Figure 1:**
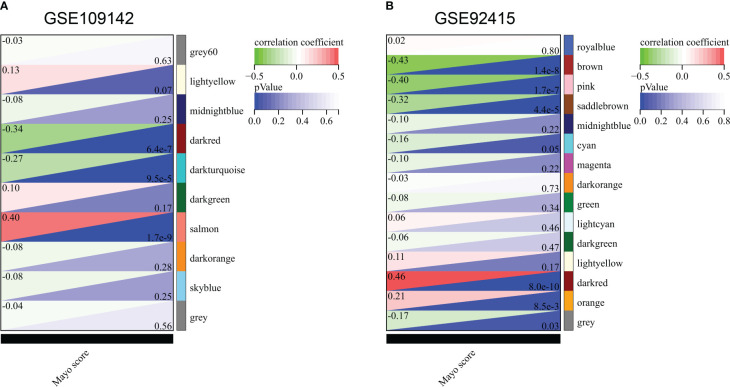
Correlation of gene co-expression modules with Mayo score in GSE109142 cohort **(A)** and GSE92415 cohort **(B)**.

In GSE109142 cohort, the soft threshold for network construction was set to 22 (**Supplementary Figures 3A, B**). In WGCNA analysis, sample clustering was performed based on gene expression patterns to detect outliers (**Supplementary Figure 3C**). Then, 9 gene modules in GSE109142 cohort were identified (**Supplementary Figures 3D, E**; **Supplementary Table 1**). The MEs of modules were utilized to evaluated Pearson’s correlation coefficients between the modules and Mayo score. Then, we identified the salmon module as the most tightly module linked with Mayo score in GSE109142 (Pearson’s correlation r = 0.40, *p* < 0.0001; **Figure 1A**). There were 1131 genes included in the salmon module (**Supplementary Figure 3E**). Subsequently, we screened 398 distinct hub genes in the salmon module based on the criteria of |MM| > 0.8 and |GS| > 0.1 (**Supplementary Table 2**).

In GSE92415 cohort, the soft threshold for network construction was set to 12 (**Supplementary Figures 4A, B**). Sample clustering in GSE92415 was also performed and shown in **Supplementary Figure 4C**. A total 14 gene modules were identified (**Supplementary Figures 4D, E**; **Supplementary Table 3**). The dark red module was the most related module with Mayo score in GSE92415 (Pearson’s correlation r = 0.46, *p* < 0.0001; **Figure 1B**). A total 375 hub genes were obtained in the dark red module (**Supplementary Table 4**).

**Figure 2:**
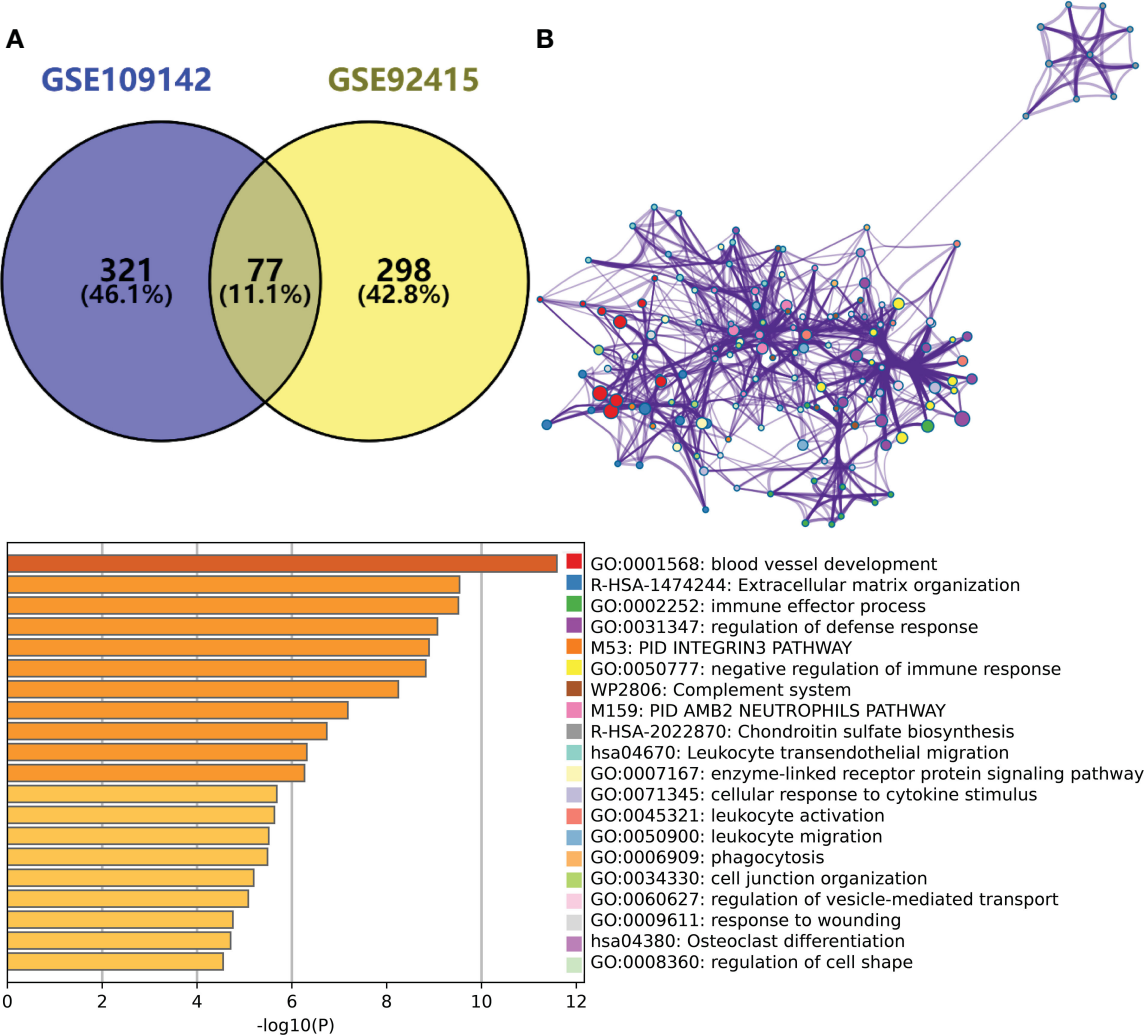
**(A)** Venn plot showing the intersection between the hub genes of GSE109142 cohort and GSE92415 cohort. **(B)** Metascape enrichment analysis results of the hub genes common to GSE109142 cohort and GSE92415 cohort (n=77).

By taking the intersection of the hub gene set in GSE109142 and GSE92415, a total 77 Mayo score-related genes were identified (**Figure 2A**). Top 20 enriched pathways of these Mayo score-related genes were revealed by Metascape analysis. These Mayo score-related genes were primarily involved in blood vessel development, immunomodulatory and inflammatory reactions (**Figure 2B**).

### Key gene signatures of high-Mayo score patients revealed by Limma and random forest analysis

**Figure 3:**
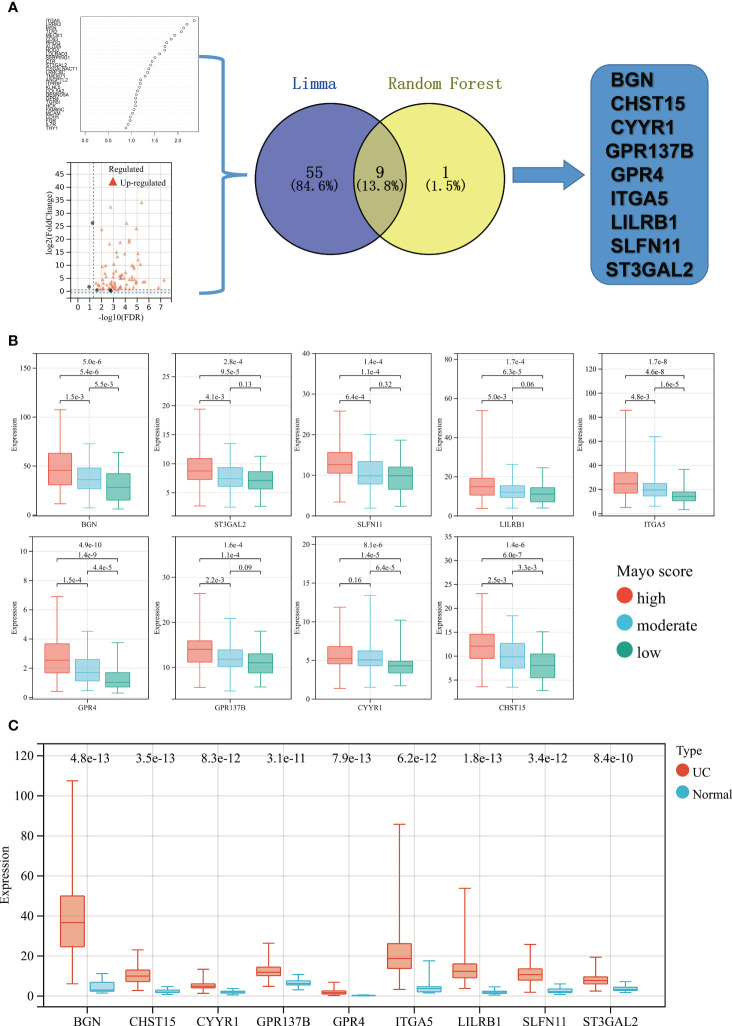
**(A)** Flowchart of HMGSs screening and selection process. **(B)** Based on upper and lower quartiles of the set of Mayo scores in GSE109142 cohort, UC patients were stratified to high- (red), moderate- (blue), and low- (green) Mayo score groups. Boxplots showing the expression levels of the 9 HMGSs across different Mayo score group. **(C)** Boxplots showing the expression levels of the 9 HMGSs in UC intestinal samples (red) and normal intestinal samples (blue).

Limma and random forest analysis was used to identify the high-Mayo score related key gene signatures (HMGSs) form the 77 Mayo score-related genes. A total 64 of 77 Mayo score-related genes were highly expressed in high-Mayo score patients. Besides, ten key genes were identified based random forest algorithm. Venn diagram showed the intersection of results of Limma and random forest analysis. Then, 9 HMGSs were screened out, including BGN,CHST15,CYYR1,GPR137B,GPR4,ITGA5,LILRB1,SLFN11 and ST3GAL2 (**Figure 3A**).

All the HMGSs were significantly up-regulated in the UC patients with high-Mayo scores and down-regulated in the UC patients with low-Mayo scores (*p* < 0.001; **Figure 3B**). In addition, the expression levels of these HMGSs were significantly higher in the UC samples compared to normal colon mucosa tissue (*p* < 0.0001; **Figure 3C**). The PCA,UMAP and tSNE analysis grouped UC samples separately from the normal healthy controls suggesting that the HMGSs was distinctive genomic signatures of the colon mucosa in UC. Furthermore, ROC analysis revealed that the overall characteristic portraits of HMGSs can be an excellent predictive indicator in the diagnosis of UC (**Supplementary Figure 5**).

### Validation of HMGSs for UC patients with high Mayo scores

In validation set (GSE92415), Spearman correlation indicated that all the 9 HMGSs were significantly positively correlated with the Mayo score, especially GPR4 (Rho=0.520; *p*<0.0001; **Supplementary Figure 6A**). The HMGSs were significantly up-regulated in UC patients with high Mayo scores in GSE92415 (*p*<0.01; **Supplementary Figure 6B**). In addition, HMGSs were significantly up-regulated in UC samples compared to normal colon mucosa tissue in GSE92415 (*p*<0.0001; **Supplementary Figure 6C**). ROC analysis suggested that HMGSs can be a predictive indicator in the diagnosis of UC patients with high Mayo scores (**Supplementary Figure 7**).

In another independent validation dataset (GSE73661), the expression levels of HMGSs were significantly higher in UC patients with higher mayo endoscopic scores (**Supplementary Figure 8A**). Lower expression levels of HMGSs were observed in non-UC tissues compared to UC tissues (**Supplementary Figure 8B**). ROC analysis suggested a good diagnostic ability of HMGSs for high mayo endoscopic score (2-3; **Supplementary Figure 8C**).

### A novel typing scheme uncover the disease severity and treatment outcomes of UC

**Figure 4:**
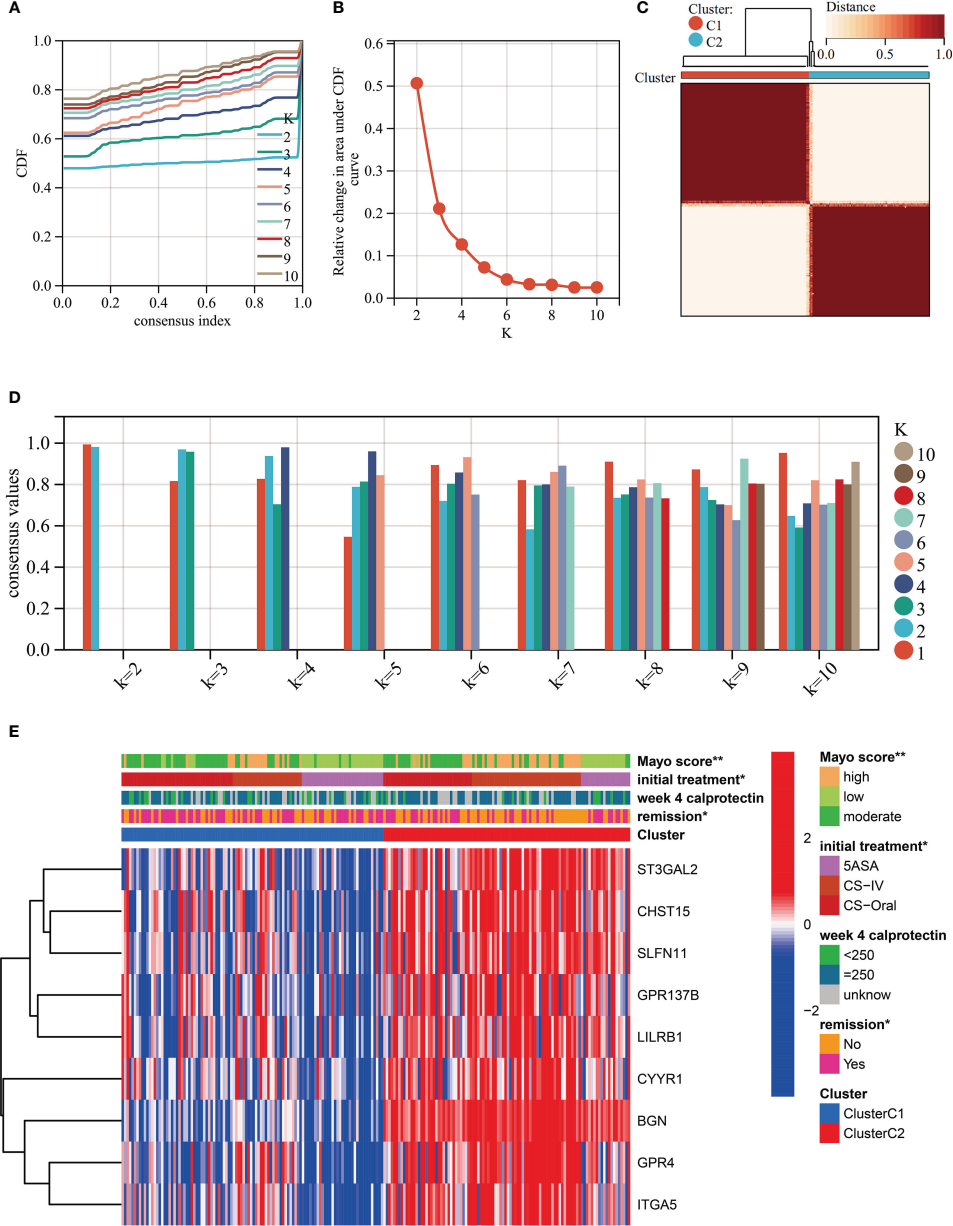
Unsupervised clustering performed in training dataset (GSE109142) based on the 9 HMGSs. **(A)** Consensus clustering cumulative distribution function (CDF) for k = 2-10. **(B)** Relative change in the area under the CDF curve (k = 2-10). **(C)** Consensus clustering matrix for k=2. **(D)** Cluster consensus values for k = 2-10. **(E)** Heatmap for the normalized expression of the 9 HMGSs.

**Figure 5:**
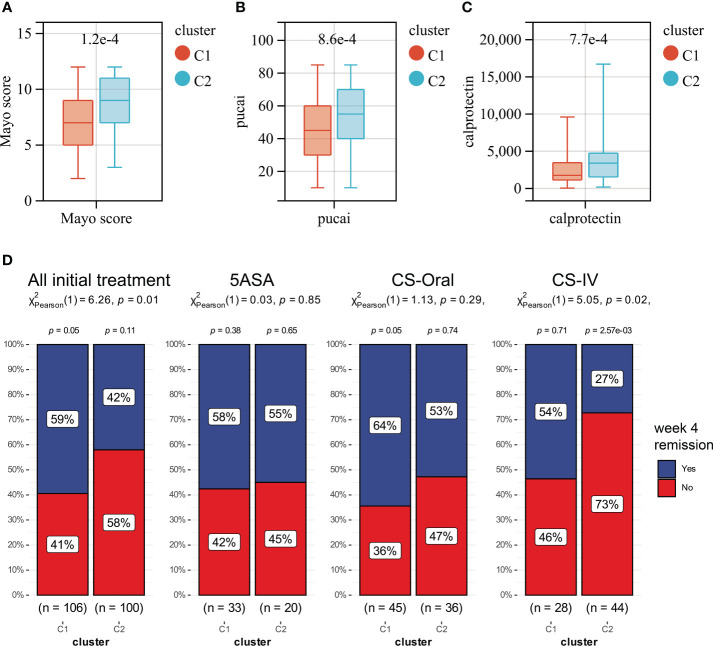
Boxplots showing the levels of Mayo score **(A)**, Pucai score **(B)** and fecal calprotectin **(C)** in cluster C1 (red) and cluster C2 (blue). **(D)** The distribution of patients who responded or did not respond to different treatments in Clusters C1 and C2.

Unsupervised clustering was performed in GSE109142 using R Package “ConsensusCluster Plus” based on the 9 HMGSs. The optimal number of clusters was determined using the empirical CDF plot (**Figures 4A, B**). On the basis of the consensus scores, the CDF curve achieved the best partition efficiency when k = 2 (**Figures 4C, D**). We therefore divided the UC patients into different molecular subtypes (cluster C1 and cluster C2). The heatmap demonstrated the distinct gene expression patterns of HMGSs between the different clusters (**Figure 4E**). Expression level of HMGSs in cluster C2 were higher than those in the cluster C1. UC patients in cluster C2 had higher levels of Mayo score, Pucai score and fecal calprotectin, suggesting a higher disease severity (**Figures 5A–C**). In GSE109142 cohort, 53 patients received 5-aminosalicylic acid (5ASA) treatment, 81 received oral corticosteroids (CS-Oral) treatment, 72 received intravenous corticosteroids (CS-IV) treatment. Symptoms were reassessed after 4 weeks of initial treatment. Chi-square test indicated that the proportion of patients with global symptom relief after initial treatment was higher in cluster C1 then that in cluster C2 (59% *vs*. 42%, *p*=0.01; **Figure 5D**). Additionally, patients of cluster C1 were more likely to derive benefit from CS-IV treatment (54% *vs*. 27%, *p*=0.02; **Figure 5D**). We carried out subsequent analyses to experimentally test whether our molecular typing scheme predicting CS-IV sensitivity is rooted in the variation of disease severity. We initially conducted ROC analysis and identified that disease severity index, Mayo score, lacks significant predictive capability towards CS-IV treatment responsiveness, with AUC=0.44(95% CI: 0.30-0.57). Furthermore, we stratified all patients receiving CS-IV treatment into high-Mayo score and low-Mayo score groups according to the median value of Mayo score (10). Subsequently, chi-square test revealed no significant difference between the proportions of patients responding to CS-IV treatment in the high-Mayo score group and the low-Mayo score group (p=0.4628). Thus, we infer that the predictive ability of our established molecular typing scheme for CS-IV treatment responsiveness is relatively independent of disease severity.

Further GSEA analysis was perform to investigate the reason for the difference of disease severity and treatment outcomes between cluster C1 and cluster C2. Several energy metabolism-associated signaling pathways were significantly up-regulated in cluster C1, including the oxidative phosphorylation, ascorbate and aldarate metabolism, Parkinson’s disease, pentose and glucuronate interconversions and citrate cycle TCA cycle pathways (**Supplementary Figure 9A**). The cluster C2 was enriched in ECM receptor interaction, neuroactive ligand receptor interaction, cell adhesion molecules cams, hedgehog signaling pathway and basal cell carcinoma pathway (**Supplementary Figure 9B**). Furthermore, 15 of the 24 measured immune cell infiltration was significantly different between cluster C1 and cluster C2 (**Supplementary Figure 9C**; **Supplementary Table 5**). The most prominent difference is the higher number of infiltrating CD 4^+^ T cells in cluster C2.

### Discovery of potential drugs by computational methods

**Figure 6:**
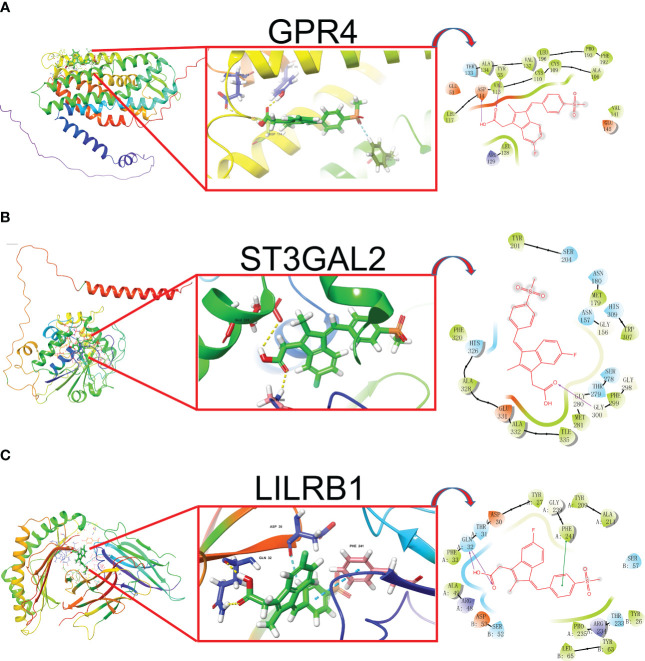
The best docked position of Exisulind inside GPR4 **(A)**, ST3GAL2 **(B)** and LILRB1 protein **(C)**.

In our study, we input the top 1000 DEGs (500 up-regulated and 500 down-regulated genes) between high- and low-Mayo score group into the “XSum” algorithm to perform cMap analysis. Then, cMap analysis revealed that Exisulind has the minimum XSum scores (**Supplementary Table 6**). Chemical structure formulae of Exisulind was shown in **Table 1**. Therefore, Exisulind was identified as the potential small molecular compounds to reverse the high Mayo score. In other words, Exisulind had the potential to attenuate the severity of UC and delay the disease progression. To further predict whether Exisulind could be a direct inhibitor for HMGSs, molecular docking was we performed based on the Schrodinger software. Exisulind showed best binding affinities for GPR4, ST3GAL2 and LILRB1 with the docking glide scores of –7.400 kcal/mol, –7.191 kcal/mol and –6.721 kcal/mol, respectively (**Figures 6A–C**). In the present study, we employed RT-qPCR to validate the gene expression levels of GPR4, ST3GAL2, and LILRB1 in the inflamed intestine of UC patients. Consistent with our previous findings, upregulation of GPR4, ST3GAL2, and LILRB1 was observed in the inflamed intestine of UC patients (n=4) compared to normal intestinal tissue (n=4), laying the foundation for considering them as potential therapeutic targets for UC (**Supplementary Figure 10**). Therefore, partial validation of Exisulind’s potential for anti-UC activity was established by its favorable molecular docking poses with the above-mentioned three genes. The docking glide scores between Exisulind and CHST15, CYYR1, ITGA5, SLFN11, GPR137B and BGN protein were -4.582 kcal/mol, -4.496 kcal/mol, -5.484 kcal/mol, -4.571 kcal/mol, -4.784 kcal/mol and -3.740 kcal/mol, respectively. In summary, Exisulind was a potential therapeutic agent for the treatment of UC.

**Table 1:** Chemical structure formulae of Exisulind.

Title	Description
PubChem CID	5472495
Structure	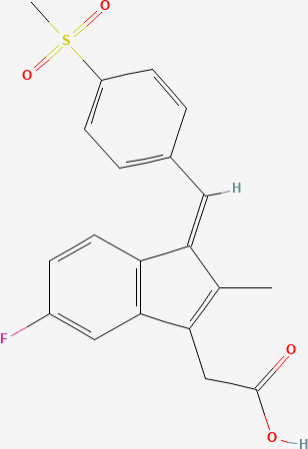
Molecular Formula	C_20_H_17_FO_4_S
Synonyms	Exisulind Sulindac sulfone Aptosyn 59973-80-7 Prevatec
Molecular Weight	372.4

### Exploration of environmental toxin exposures with potential to impact the severity of UC

We explored all potential Environmental Toxin Exposures that may impact the expression levels of HMGSs by leveraging the CTD database. Subsequently, we have acquired a total of 110 different types of Environmental Toxin Exposures that could affect the expression level or methylation state of HMGSs, showing in **Table 2**. Thus, these Environmental Toxin Exposures have the potential to modulate the severity of UC, an effect that is mediated by the intermediary factors HMGSs. Hence, avoiding exposure to these toxins might facilitate an improvement in therapeutic responsiveness among UC patients.

**Table 2:** The interaction between environmental toxin exposure and HMGSs

Chemical Name	Gene Symbol	Organism	Interaction	ReferencesPubMedID
Benzo(a)pyrene	GPR4	Homo sapiens	Benzo(a)pyrene affects the methylation of GPR4 promoter	27901495
Benzo(a)pyrene	GPR4	Homo sapiens	Benzo(a)pyrene results in decreased expression of GPR4 mRNA	22316170
Benzo(a)pyrene	GPR4	Homo sapiens	Benzo(a)pyrene results in increased methylation of GPR4 3' UTR	27901495
Benzo(a)pyrene	GPR4	Homo sapiens	Benzo(a)pyrene results in increased methylation of GPR4 5' UTR	27901495
bisphenol A	GPR4	Homo sapiens	bisphenol A results in decreased expression of GPR4 mRNA	31715268
butyraldehyde	GPR4	Homo sapiens	butyraldehyde results in increased expression of GPR4 mRNA	26079696
Cadmium	GPR4	Homo sapiens	Cadmium results in decreased expression of GPR4 mRNA	24376830
cobaltous chloride	GPR4	Homo sapiens	cobaltous chloride results in decreased expression of GPR4 mRNA	19320972
Nickel	GPR4	Homo sapiens	Nickel results in increased expression of GPR4 mRNA	25583101
nickel sulfate	GPR4	Homo sapiens	nickel sulfate results in decreased expression of GPR4 mRNA	22714537
Oxygen	GPR4	Homo sapiens	Oxygen deficiency results in increased expression of GPR4 protein	33161135
Smoke	GPR4	Homo sapiens	Smoke results in decreased expression of GPR4 mRNA	34520756
Sugars	GPR4	Homo sapiens	[Anti-Inflammatory Agents binds to and results in decreased activity of GPR4 protein] inhibits the reaction [[Biological Factors binds to Sugars] which results in increased expression of IL1B mRNA]	32370492
Sugars	GPR4	Homo sapiens	[Anti-Inflammatory Agents binds to and results in decreased activity of GPR4 protein] inhibits the reaction [[Biological Factors binds to Sugars] which results in increased expression of IL1B protein]	32370492
Sugars	GPR4	Homo sapiens	[Anti-Inflammatory Agents binds to and results in decreased activity of GPR4 protein] inhibits the reaction [[Biological Factors binds to Sugars] which results in increased expression of IL6 mRNA]	32370492
Sugars	GPR4	Homo sapiens	[Anti-Inflammatory Agents binds to and results in decreased activity of GPR4 protein] inhibits the reaction [[Biological Factors binds to Sugars] which results in increased expression of IL6 protein]	32370492
Sugars	GPR4	Homo sapiens	[Anti-Inflammatory Agents binds to and results in decreased activity of GPR4 protein] inhibits the reaction [[Biological Factors binds to Sugars] which results in increased expression of MMP13 mRNA]	32370492
Sugars	GPR4	Homo sapiens	[Anti-Inflammatory Agents binds to and results in decreased activity of GPR4 protein] inhibits the reaction [[Biological Factors binds to Sugars] which results in increased expression of MMP13 protein]	32370492
Sugars	GPR4	Homo sapiens	[Anti-Inflammatory Agents binds to and results in decreased activity of GPR4 protein] inhibits the reaction [[Biological Factors binds to Sugars] which results in increased expression of MMP3 mRNA]	32370492
Sugars	GPR4	Homo sapiens	[Anti-Inflammatory Agents binds to and results in decreased activity of GPR4 protein] inhibits the reaction [[Biological Factors binds to Sugars] which results in increased expression of MMP3 protein]	32370492
Sugars	GPR4	Homo sapiens	[Anti-Inflammatory Agents binds to and results in decreased activity of GPR4 protein] inhibits the reaction [[Biological Factors binds to Sugars] which results in increased expression of NOS2 mRNA]	32370492
Sugars	GPR4	Homo sapiens	[Anti-Inflammatory Agents binds to and results in decreased activity of GPR4 protein] inhibits the reaction [[Biological Factors binds to Sugars] which results in increased expression of NOS2 protein]	32370492
Sugars	GPR4	Homo sapiens	[Anti-Inflammatory Agents binds to and results in decreased activity of GPR4 protein] inhibits the reaction [[Biological Factors binds to Sugars] which results in increased expression of PTGS2 mRNA]	32370492
Sugars	GPR4	Homo sapiens	[Anti-Inflammatory Agents binds to and results in decreased activity of GPR4 protein] inhibits the reaction [[Biological Factors binds to Sugars] which results in increased expression of PTGS2 protein]	32370492
Sugars	GPR4	Homo sapiens	[Anti-Inflammatory Agents binds to and results in decreased activity of GPR4 protein] inhibits the reaction [[Biological Factors binds to Sugars] which results in increased expression of TNF mRNA]	32370492
Sugars	GPR4	Homo sapiens	[Anti-Inflammatory Agents binds to and results in decreased activity of GPR4 protein] inhibits the reaction [[Biological Factors binds to Sugars] which results in increased expression of TNF protein]	32370492
Sugars	GPR4	Homo sapiens	[Biological Factors binds to Sugars] which results in increased expression of GPR4 mRNA	32370492
Sugars	GPR4	Homo sapiens	[Biological Factors binds to Sugars] which results in increased expression of GPR4 protein	32370492
tert-Butylhydroperoxide	GPR4	Homo sapiens	tert-Butylhydroperoxide results in increased expression of GPR4 mRNA	15336504
4-(5-benzo(1,3)dioxol-5-yl-4-pyridin-2-yl-1H-imidazol-2-yl)benzamide	CYYR1	Homo sapiens	[NOG protein co-treated with methylmercuric chloride co-treated with dorsomorphin co-treated with 4-(5-benzo(1,3)dioxol-5-yl-4-pyridin-2-yl-1H-imidazol-2-yl)benzamide] results in increased expression of CYYR1 mRNA	27188386
Aflatoxin B1	CYYR1	Homo sapiens	Aflatoxin B1 results in decreased methylation of CYYR1 gene	27153756
Arsenic	CYYR1	Homo sapiens	[sodium arsenate results in increased abundance of Arsenic] which results in decreased expression of CYYR1 mRNA	32525701
arsenite	CYYR1	Homo sapiens	arsenite results in decreased expression of CYYR1 mRNA	23974009
Benzo(a)pyrene	CYYR1	Homo sapiens	Benzo(a)pyrene results in increased methylation of CYYR1 promoter	27901495
Cadmium	CYYR1	Homo sapiens	Cadmium results in decreased expression of CYYR1 mRNA	23369406
cobaltous chloride	CYYR1	Homo sapiens	cobaltous chloride results in decreased expression of CYYR1 mRNA	19320972
dorsomorphin	CYYR1	Homo sapiens	[NOG protein co-treated with methylmercuric chloride co-treated with dorsomorphin co-treated with 4-(5-benzo(1,3)dioxol-5-yl-4-pyridin-2-yl-1H-imidazol-2-yl)benzamide] results in increased expression of CYYR1 mRNA	27188386
Ethanol	CYYR1	Homo sapiens	Ethanol results in decreased expression of CYYR1 mRNA	23378141
methylmercuric chloride	CYYR1	Homo sapiens	methylmercuric chloride results in increased expression of CYYR1 mRNA	23179753|26272509
methylmercuric chloride	CYYR1	Homo sapiens	[NOG protein co-treated with methylmercuric chloride co-treated with dorsomorphin co-treated with 4-(5-benzo(1,3)dioxol-5-yl-4-pyridin-2-yl-1H-imidazol-2-yl)benzamide] results in increased expression of CYYR1 mRNA	27188386
Silicon Dioxide	CYYR1	Homo sapiens	Silicon Dioxide analog results in increased expression of CYYR1 mRNA	23806026
sodium arsenate	CYYR1	Homo sapiens	[sodium arsenate results in increased abundance of Arsenic] which results in decreased expression of CYYR1 mRNA	32525701
Tobacco Smoke Pollution	CYYR1	Homo sapiens	Tobacco Smoke Pollution results in decreased expression of CYYR1 mRNA	30291989|33660061
Aflatoxin B1	ST3GAL2	Homo sapiens	Aflatoxin B1 results in decreased methylation of ST3GAL2 intron	30157460
Arsenic	ST3GAL2	Homo sapiens	Arsenic affects the methylation of ST3GAL2 gene	25304211
Arsenic Trioxide	ST3GAL2	Homo sapiens	Arsenic Trioxide results in decreased expression of ST3GAL2 mRNA	20458559
Arsenic Trioxide	ST3GAL2	Homo sapiens	Arsenic Trioxide results in increased expression of ST3GAL2 mRNA	20458559
Benzo(a)pyrene	ST3GAL2	Homo sapiens	Benzo(a)pyrene results in increased methylation of ST3GAL2 5' UTR	27901495
Copper Sulfate	ST3GAL2	Homo sapiens	Copper Sulfate results in decreased expression of ST3GAL2 mRNA	19549813
Diazinon	ST3GAL2	Homo sapiens	Diazinon results in increased methylation of ST3GAL2 gene	22964155
dicrotophos	ST3GAL2	Homo sapiens	dicrotophos results in increased expression of ST3GAL2 mRNA	28302478
Methyl Methanesulfonate	ST3GAL2	Homo sapiens	Methyl Methanesulfonate results in decreased expression of ST3GAL2 mRNA	23649840
Smoke	ST3GAL2	Homo sapiens	Smoke results in decreased expression of ST3GAL2 mRNA	34520756
Tobacco Smoke Pollution	ST3GAL2	Homo sapiens	Tobacco Smoke Pollution results in decreased expression of ST3GAL2 mRNA	33660061
Vehicle Emissions	ST3GAL2	Homo sapiens	Vehicle Emissions results in decreased methylation of ST3GAL2 gene	25487561
1-Butanol	CHST15	Homo sapiens	[[Gasoline co-treated with 1-Butanol] results in increased abundance of [Particulate Matter co-treated with Polycyclic Aromatic Hydrocarbons]] which results in decreased expression of CHST15 mRNA	29432896
4-(4-((5-(4,5-dimethyl-2-nitrophenyl)-2-furanyl)methylene)-4,5-dihydro-3-methyl-5-oxo-1H-pyrazol-1-yl)benzoic acid	CHST15	Homo sapiens	4-(4-((5-(4,5-dimethyl-2-nitrophenyl)-2-furanyl)methylene)-4,5-dihydro-3-methyl-5-oxo-1H-pyrazol-1-yl)benzoic acid results in increased expression of CHST15 mRNA	26191083
4-(5-benzo(1,3)dioxol-5-yl-4-pyridin-2-yl-1H-imidazol-2-yl)benzamide	CHST15	Homo sapiens	[NOG protein co-treated with entinostat co-treated with dorsomorphin co-treated with 4-(5-benzo(1,3)dioxol-5-yl-4-pyridin-2-yl-1H-imidazol-2-yl)benzamide] results in increased expression of CHST15 mRNA	27188386
4-(5-benzo(1,3)dioxol-5-yl-4-pyridin-2-yl-1H-imidazol-2-yl)benzamide	CHST15	Homo sapiens	[NOG protein co-treated with Panobinostat co-treated with dorsomorphin co-treated with 4-(5-benzo(1,3)dioxol-5-yl-4-pyridin-2-yl-1H-imidazol-2-yl)benzamide] results in increased expression of CHST15 mRNA	27188386
4-(5-benzo(1,3)dioxol-5-yl-4-pyridin-2-yl-1H-imidazol-2-yl)benzamide	CHST15	Homo sapiens	[NOG protein co-treated with Phenylmercuric Acetate co-treated with dorsomorphin co-treated with 4-(5-benzo(1,3)dioxol-5-yl-4-pyridin-2-yl-1H-imidazol-2-yl)benzamide] results in increased expression of CHST15 mRNA	27188386
4-(5-benzo(1,3)dioxol-5-yl-4-pyridin-2-yl-1H-imidazol-2-yl)benzamide	CHST15	Homo sapiens	[NOG protein co-treated with trichostatin A co-treated with dorsomorphin co-treated with 4-(5-benzo(1,3)dioxol-5-yl-4-pyridin-2-yl-1H-imidazol-2-yl)benzamide] results in increased expression of CHST15 mRNA	27188386
4-(5-benzo(1,3)dioxol-5-yl-4-pyridin-2-yl-1H-imidazol-2-yl)benzamide	CHST15	Homo sapiens	[NOG protein co-treated with Valproic Acid co-treated with dorsomorphin co-treated with 4-(5-benzo(1,3)dioxol-5-yl-4-pyridin-2-yl-1H-imidazol-2-yl)benzamide] results in increased expression of CHST15 mRNA	27188386
7,8-Dihydro-7,8-dihydroxybenzo(a)pyrene 9,10-oxide	CHST15	Homo sapiens	7,8-Dihydro-7,8-dihydroxybenzo(a)pyrene 9,10-oxide results in decreased expression of CHST15 mRNA	19150397|20382639
7,8-Dihydro-7,8-dihydroxybenzo(a)pyrene 9,10-oxide	CHST15	Homo sapiens	7,8-Dihydro-7,8-dihydroxybenzo(a)pyrene 9,10-oxide results in increased expression of CHST15 mRNA	26238291
9,10-dihydro-9,10-dihydroxybenzo(a)pyrene	CHST15	Homo sapiens	9,10-dihydro-9,10-dihydroxybenzo(a)pyrene results in decreased expression of CHST15 mRNA	26238291
Air Pollutants, Occupational	CHST15	Homo sapiens	Air Pollutants, Occupational results in decreased expression of CHST15 mRNA	23195993
arsenite	CHST15	Homo sapiens	arsenite results in increased methylation of CHST15 promoter	23974009
Benzo(a)pyrene	CHST15	Homo sapiens	Benzo(a)pyrene affects the expression of CHST15 mRNA	22316170
Benzo(a)pyrene	CHST15	Homo sapiens	Benzo(a)pyrene affects the methylation of CHST15 intron	30157460
Benzo(a)pyrene	CHST15	Homo sapiens	Benzo(a)pyrene results in decreased expression of CHST15 mRNA	20106945|21632981|26238291
Benzo(a)pyrene	CHST15	Homo sapiens	Benzo(a)pyrene results in increased expression of CHST15 mRNA	32234424
bisphenol A	CHST15	Homo sapiens	bisphenol A affects the expression of CHST15 mRNA	30903817
bisphenol A	CHST15	Homo sapiens	[bisphenol A co-treated with Fulvestrant] results in increased methylation of CHST15 gene	31601247
bisphenol A	CHST15	Homo sapiens	bisphenol A results in decreased methylation of CHST15 gene	31601247
Copper Sulfate	CHST15	Homo sapiens	Copper Sulfate results in decreased expression of CHST15 mRNA	19549813
Diethylhexyl Phthalate	CHST15	Homo sapiens	Diethylhexyl Phthalate results in decreased expression of CHST15 mRNA	28412506
dorsomorphin	CHST15	Homo sapiens	[NOG protein co-treated with entinostat co-treated with dorsomorphin co-treated with 4-(5-benzo(1,3)dioxol-5-yl-4-pyridin-2-yl-1H-imidazol-2-yl)benzamide] results in increased expression of CHST15 mRNA	27188386
dorsomorphin	CHST15	Homo sapiens	[NOG protein co-treated with Panobinostat co-treated with dorsomorphin co-treated with 4-(5-benzo(1,3)dioxol-5-yl-4-pyridin-2-yl-1H-imidazol-2-yl)benzamide] results in increased expression of CHST15 mRNA	27188386
dorsomorphin	CHST15	Homo sapiens	[NOG protein co-treated with Phenylmercuric Acetate co-treated with dorsomorphin co-treated with 4-(5-benzo(1,3)dioxol-5-yl-4-pyridin-2-yl-1H-imidazol-2-yl)benzamide] results in increased expression of CHST15 mRNA	27188386
dorsomorphin	CHST15	Homo sapiens	[NOG protein co-treated with trichostatin A co-treated with dorsomorphin co-treated with 4-(5-benzo(1,3)dioxol-5-yl-4-pyridin-2-yl-1H-imidazol-2-yl)benzamide] results in increased expression of CHST15 mRNA	27188386
dorsomorphin	CHST15	Homo sapiens	[NOG protein co-treated with Valproic Acid co-treated with dorsomorphin co-treated with 4-(5-benzo(1,3)dioxol-5-yl-4-pyridin-2-yl-1H-imidazol-2-yl)benzamide] results in increased expression of CHST15 mRNA	27188386
Ethanol	CHST15	Homo sapiens	[[Gasoline co-treated with Ethanol] results in increased abundance of [Particulate Matter co-treated with Polycyclic Aromatic Hydrocarbons]] which results in decreased expression of CHST15 mRNA	29432896
Formaldehyde	CHST15	Homo sapiens	Formaldehyde results in decreased expression of CHST15 mRNA	20655997
Formaldehyde	CHST15	Homo sapiens	Formaldehyde results in increased expression of CHST15 mRNA	23649840
Gasoline	CHST15	Homo sapiens	[[Gasoline co-treated with 1-Butanol] results in increased abundance of [Particulate Matter co-treated with Polycyclic Aromatic Hydrocarbons]] which results in decreased expression of CHST15 mRNA	29432896
Gasoline	CHST15	Homo sapiens	[[Gasoline co-treated with Ethanol] results in increased abundance of [Particulate Matter co-treated with Polycyclic Aromatic Hydrocarbons]] which results in decreased expression of CHST15 mRNA	29432896
Gasoline	CHST15	Homo sapiens	[Gasoline results in increased abundance of [Particulate Matter co-treated with Polycyclic Aromatic Hydrocarbons]] which results in decreased expression of CHST15 mRNA	29432896
Hydrogen Peroxide	CHST15	Homo sapiens	Hydrogen Peroxide affects the expression of CHST15 mRNA	23410634
Mustard Gas	CHST15	Homo sapiens	Mustard Gas results in decreased expression of CHST15 mRNA	12884408
Nickel	CHST15	Homo sapiens	Nickel results in decreased expression of CHST15 mRNA	23195993
nickel sulfate	CHST15	Homo sapiens	nickel sulfate results in decreased expression of CHST15 mRNA	22714537
Particulate Matter	CHST15	Homo sapiens	[[Gasoline co-treated with 1-Butanol] results in increased abundance of [Particulate Matter co-treated with Polycyclic Aromatic Hydrocarbons]] which results in decreased expression of CHST15 mRNA	29432896
Particulate Matter	CHST15	Homo sapiens	[[Gasoline co-treated with Ethanol] results in increased abundance of [Particulate Matter co-treated with Polycyclic Aromatic Hydrocarbons]] which results in decreased expression of CHST15 mRNA	29432896
Particulate Matter	CHST15	Homo sapiens	[Gasoline results in increased abundance of [Particulate Matter co-treated with Polycyclic Aromatic Hydrocarbons]] which results in decreased expression of CHST15 mRNA	29432896
Phenylmercuric Acetate	CHST15	Homo sapiens	[NOG protein co-treated with Phenylmercuric Acetate co-treated with dorsomorphin co-treated with 4-(5-benzo(1,3)dioxol-5-yl-4-pyridin-2-yl-1H-imidazol-2-yl)benzamide] results in increased expression of CHST15 mRNA	27188386
Phenylmercuric Acetate	CHST15	Homo sapiens	Phenylmercuric Acetate results in increased expression of CHST15 mRNA	26272509
Polycyclic Aromatic Hydrocarbons	CHST15	Homo sapiens	[[Gasoline co-treated with 1-Butanol] results in increased abundance of [Particulate Matter co-treated with Polycyclic Aromatic Hydrocarbons]] which results in decreased expression of CHST15 mRNA	29432896
Polycyclic Aromatic Hydrocarbons	CHST15	Homo sapiens	[[Gasoline co-treated with Ethanol] results in increased abundance of [Particulate Matter co-treated with Polycyclic Aromatic Hydrocarbons]] which results in decreased expression of CHST15 mRNA	29432896
Polycyclic Aromatic Hydrocarbons	CHST15	Homo sapiens	[Gasoline results in increased abundance of [Particulate Matter co-treated with Polycyclic Aromatic Hydrocarbons]] which results in decreased expression of CHST15 mRNA	29432896
potassium chromate(VI)	CHST15	Homo sapiens	[potassium chromate(VI) co-treated with epigallocatechin gallate] results in decreased expression of CHST15 mRNA	22079256
potassium chromate(VI)	CHST15	Homo sapiens	potassium chromate(VI) results in decreased expression of CHST15 mRNA	22079256
Smoke	CHST15	Homo sapiens	Smoke results in decreased expression of CHST15 mRNA	34520756
tert-Butylhydroperoxide	CHST15	Homo sapiens	tert-Butylhydroperoxide affects the expression of CHST15 mRNA	23410634
Tetrachlorodibenzodioxin	CHST15	Homo sapiens	Tetrachlorodibenzodioxin results in decreased expression of CHST15 mRNA	20106945|21632981|26238291
Tobacco Smoke Pollution	CHST15	Homo sapiens	Tobacco Smoke Pollution results in decreased expression of CHST15 mRNA	28065790
Tobacco Smoke Pollution	CHST15	Homo sapiens	Tobacco Smoke Pollution results in increased expression of CHST15 mRNA	33660061
Triclosan	CHST15	Homo sapiens	Triclosan results in decreased expression of CHST15 mRNA	30510588
tris(1,3-dichloro-2-propyl)phosphate	CHST15	Homo sapiens	tris(1,3-dichloro-2-propyl)phosphate results in decreased expression of CHST15 mRNA	26179874
Urethane	CHST15	Homo sapiens	Urethane results in decreased expression of CHST15 mRNA	28818685
Vanadates	CHST15	Homo sapiens	Vanadates results in increased expression of CHST15 mRNA	22714537
4-(5-benzo(1,3)dioxol-5-yl-4-pyridin-2-yl-1H-imidazol-2-yl)benzamide	SLFN11	Homo sapiens	[NOG protein co-treated with Valproic Acid co-treated with dorsomorphin co-treated with 4-(5-benzo(1,3)dioxol-5-yl-4-pyridin-2-yl-1H-imidazol-2-yl)benzamide] results in increased expression of SLFN11 mRNA	27188386
7,8-Dihydro-7,8-dihydroxybenzo(a)pyrene 9,10-oxide	SLFN11	Homo sapiens	7,8-Dihydro-7,8-dihydroxybenzo(a)pyrene 9,10-oxide results in decreased expression of SLFN11 mRNA	20382639
Benzene	SLFN11	Homo sapiens	Benzene results in increased expression of SLFN11 mRNA	15929907
Benzo(a)pyrene	SLFN11	Homo sapiens	Benzo(a)pyrene affects the methylation of SLFN11 promoter	27901495
Benzo(a)pyrene	SLFN11	Homo sapiens	Benzo(a)pyrene results in decreased methylation of SLFN11 5' UTR	27901495
bisphenol A	SLFN11	Homo sapiens	bisphenol A results in decreased methylation of SLFN11 gene	31601247
cobaltous chloride	SLFN11	Homo sapiens	cobaltous chloride results in decreased expression of SLFN11 mRNA	19320972
Copper	SLFN11	Homo sapiens	[Disulfiram binds to Copper] which results in decreased expression of SLFN11 mRNA	24690739
dorsomorphin	SLFN11	Homo sapiens	[NOG protein co-treated with Valproic Acid co-treated with dorsomorphin co-treated with 4-(5-benzo(1,3)dioxol-5-yl-4-pyridin-2-yl-1H-imidazol-2-yl)benzamide] results in increased expression of SLFN11 mRNA	27188386
methylmercuric chloride	SLFN11	Homo sapiens	methylmercuric chloride results in increased expression of SLFN11 mRNA	28001369
Mustard Gas	SLFN11	Homo sapiens	Mustard Gas results in decreased expression of SLFN11 mRNA	25102026
Nickel	SLFN11	Homo sapiens	Nickel results in increased expression of SLFN11 mRNA	25583101
Polystyrenes	SLFN11	Homo sapiens	Polystyrenes results in increased expression of SLFN11 mRNA	25102311
potassium chromate(VI)	SLFN11	Homo sapiens	potassium chromate(VI) results in increased expression of SLFN11 mRNA	22714537
Tobacco Smoke Pollution	SLFN11	Homo sapiens	Tobacco Smoke Pollution results in decreased expression of SLFN11 mRNA	33660061
Vanadates	SLFN11	Homo sapiens	Vanadates results in decreased expression of SLFN11 mRNA	22714537
4-(4-((5-(4,5-dimethyl-2-nitrophenyl)-2-furanyl)methylene)-4,5-dihydro-3-methyl-5-oxo-1H-pyrazol-1-yl)benzoic acid	GPR137B	Homo sapiens	4-(4-((5-(4,5-dimethyl-2-nitrophenyl)-2-furanyl)methylene)-4,5-dihydro-3-methyl-5-oxo-1H-pyrazol-1-yl)benzoic acid results in increased expression of GPR137B mRNA	26191083
4-(5-benzo(1,3)dioxol-5-yl-4-pyridin-2-yl-1H-imidazol-2-yl)benzamide	GPR137B	Homo sapiens	[NOG protein co-treated with Phenylmercuric Acetate co-treated with dorsomorphin co-treated with 4-(5-benzo(1,3)dioxol-5-yl-4-pyridin-2-yl-1H-imidazol-2-yl)benzamide] results in increased expression of GPR137B mRNA	27188386
7,8-Dihydro-7,8-dihydroxybenzo(a)pyrene 9,10-oxide	GPR137B	Homo sapiens	7,8-Dihydro-7,8-dihydroxybenzo(a)pyrene 9,10-oxide results in increased expression of GPR137B mRNA	20382639
Aflatoxin B1	GPR137B	Homo sapiens	Aflatoxin B1 affects the methylation of GPR137B intron	30157460
Aflatoxin B1	GPR137B	Homo sapiens	Aflatoxin B1 results in decreased methylation of GPR137B gene	27153756
aflatoxin B2	GPR137B	Homo sapiens	aflatoxin B2 results in increased methylation of GPR137B intron	30157460
aristolochic acid I	GPR137B	Homo sapiens	aristolochic acid I results in decreased expression of GPR137B mRNA	33212167
Arsenic	GPR137B	Homo sapiens	Arsenic affects the methylation of GPR137B gene	25304211
Benzene	GPR137B	Homo sapiens	Benzene results in increased expression of GPR137B mRNA	19162166
Benzo(a)pyrene	GPR137B	Homo sapiens	Benzo(a)pyrene affects the methylation of GPR137B intron	30157460
Benzo(a)pyrene	GPR137B	Homo sapiens	Benzo(a)pyrene affects the methylation of GPR137B promoter	27901495
benzo(e)pyrene	GPR137B	Homo sapiens	benzo(e)pyrene results in increased methylation of GPR137B intron	30157460
bisphenol A	GPR137B	Homo sapiens	bisphenol A results in increased expression of GPR137B mRNA	29275510
Copper Sulfate	GPR137B	Homo sapiens	Copper Sulfate results in increased expression of GPR137B mRNA	19549813
dorsomorphin	GPR137B	Homo sapiens	[NOG protein co-treated with Phenylmercuric Acetate co-treated with dorsomorphin co-treated with 4-(5-benzo(1,3)dioxol-5-yl-4-pyridin-2-yl-1H-imidazol-2-yl)benzamide] results in increased expression of GPR137B mRNA	27188386
Fonofos	GPR137B	Homo sapiens	Fonofos results in decreased methylation of GPR137B promoter	22847954
Formaldehyde	GPR137B	Homo sapiens	Formaldehyde results in increased expression of GPR137B mRNA	23649840
Lead	GPR137B	Homo sapiens	Lead affects the expression of GPR137B mRNA	28903495
methylmercuric chloride	GPR137B	Homo sapiens	methylmercuric chloride results in increased expression of GPR137B mRNA	28001369
Methyl Methanesulfonate	GPR137B	Homo sapiens	Methyl Methanesulfonate results in increased expression of GPR137B mRNA	21527772
Parathion	GPR137B	Homo sapiens	Parathion results in decreased methylation of GPR137B promoter	22847954
Phenylmercuric Acetate	GPR137B	Homo sapiens	[NOG protein co-treated with Phenylmercuric Acetate co-treated with dorsomorphin co-treated with 4-(5-benzo(1,3)dioxol-5-yl-4-pyridin-2-yl-1H-imidazol-2-yl)benzamide] results in increased expression of GPR137B mRNA	27188386
Phenylmercuric Acetate	GPR137B	Homo sapiens	Phenylmercuric Acetate results in increased expression of GPR137B mRNA	26272509
Selenium	GPR137B	Homo sapiens	Selenium results in decreased expression of GPR137B mRNA	19244175
Silicon Dioxide	GPR137B	Homo sapiens	Silicon Dioxide analog results in increased expression of GPR137B mRNA	25895662
terbufos	GPR137B	Homo sapiens	terbufos results in decreased methylation of GPR137B promoter	22847954
Tobacco Smoke Pollution	GPR137B	Homo sapiens	Tobacco Smoke Pollution results in increased expression of GPR137B mRNA	28065790
tris(1,3-dichloro-2-propyl)phosphate	GPR137B	Homo sapiens	tris(1,3-dichloro-2-propyl)phosphate results in increased expression of GPR137B mRNA	26179874
Urethane	GPR137B	Homo sapiens	Urethane results in increased expression of GPR137B mRNA	28818685
vanadyl sulfate	GPR137B	Homo sapiens	vanadyl sulfate results in decreased expression of GPR137B mRNA	16330358
1-Methyl-3-isobutylxanthine	ITGA5	Homo sapiens	[INS protein co-treated with Dexamethasone co-treated with 1-Methyl-3-isobutylxanthine co-treated with Indomethacin co-treated with bis(4-hydroxyphenyl)sulfone] results in decreased expression of ITGA5 mRNA	28628672
1-Methyl-3-isobutylxanthine	ITGA5	Homo sapiens	[INS protein co-treated with Dexamethasone co-treated with 1-Methyl-3-isobutylxanthine co-treated with Indomethacin co-treated with bisphenol A] results in decreased expression of ITGA5 mRNA	28628672
1-Naphthylisothiocyanate	ITGA5	Homo sapiens	1-Naphthylisothiocyanate results in increased expression of [ITGB6 protein co-treated with ITGA5 protein]	21037076
1-Naphthylisothiocyanate	ITGA5	Homo sapiens	[1-Naphthylisothiocyanate results in increased expression of [ITGB6 protein co-treated with ITGA5 protein]] which results in increased activity of TGFB1 protein	21037076
1-Naphthylisothiocyanate	ITGA5	Homo sapiens	F2R protein promotes the reaction [1-Naphthylisothiocyanate results in increased expression of [ITGB6 protein co-treated with ITGA5 protein]]	21037076
1-Naphthylisothiocyanate	ITGA5	Homo sapiens	F3 protein promotes the reaction [1-Naphthylisothiocyanate results in increased expression of [ITGB6 protein co-treated with ITGA5 protein]]	21037076
2-(4-morpholinyl)-8-phenyl-4H-1-benzopyran-4-one	ITGA5	Homo sapiens	2-(4-morpholinyl)-8-phenyl-4H-1-benzopyran-4-one inhibits the reaction [IGF1 protein results in increased expression of and affects the localization of [ITGA5 protein binds to ITGB3 protein]]	16465378
2-methoxy-N-(3-methyl-2-oxo-1,2,3,4-tetrahydroquinazolin-6-yl)benzenesulfonamide	ITGA5	Homo sapiens	2-methoxy-N-(3-methyl-2-oxo-1,2,3,4-tetrahydroquinazolin-6-yl)benzenesulfonamide inhibits the reaction [TGFB1 protein results in increased expression of ITGA5 mRNA]	26644586
2-methoxy-N-(3-methyl-2-oxo-1,2,3,4-tetrahydroquinazolin-6-yl)benzenesulfonamide	ITGA5	Homo sapiens	2-methoxy-N-(3-methyl-2-oxo-1,2,3,4-tetrahydroquinazolin-6-yl)benzenesulfonamide results in decreased expression of ITGA5 mRNA	26644586
3-(6-methoxypyridin-3-yl)-3-(2-oxo-3-(3-(5,6,7,8-tetrahydro(1,8)naphthyridin-2-yl)propyl)imidazolidin-1-yl)propionic acid	ITGA5	Homo sapiens	3-(6-methoxypyridin-3-yl)-3-(2-oxo-3-(3-(5,6,7,8-tetrahydro(1,8)naphthyridin-2-yl)propyl)imidazolidin-1-yl)propionic acid binds to [ITGA5 protein binds to ITGB3 protein]	14561098
4-(4-((5-(4,5-dimethyl-2-nitrophenyl)-2-furanyl)methylene)-4,5-dihydro-3-methyl-5-oxo-1H-pyrazol-1-yl)benzoic acid	ITGA5	Homo sapiens	4-(4-((5-(4,5-dimethyl-2-nitrophenyl)-2-furanyl)methylene)-4,5-dihydro-3-methyl-5-oxo-1H-pyrazol-1-yl)benzoic acid results in increased expression of ITGA5 mRNA	26191083
4-hydroxy-2-nonenal	ITGA5	Homo sapiens	4-hydroxy-2-nonenal results in decreased expression of ITGA5 mRNA	12419474
Aluminum Oxide	ITGA5	Homo sapiens	[Aluminum Oxide co-treated with Magnesium] results in increased expression of ITGA5 protein	12209937
aristolochic acid I	ITGA5	Homo sapiens	aristolochic acid I results in increased expression of ITGA5 mRNA	33212167
Arsenic	ITGA5	Homo sapiens	[sodium arsenate results in increased abundance of Arsenic] which results in increased expression of ITGA5 mRNA	32525701
Atrazine	ITGA5	Homo sapiens	Atrazine inhibits the reaction [Tetradecanoylphorbol Acetate results in increased expression of ITGA5 mRNA]	24211529
Benomyl	ITGA5	Homo sapiens	Benomyl results in decreased expression of ITGA5 mRNA	25530041
Benzene	ITGA5	Homo sapiens	Benzene results in increased expression of ITGA5 mRNA	19162166
benzo(e)pyrene	ITGA5	Homo sapiens	benzo(e)pyrene results in increased methylation of ITGA5 intron	30157460
bisphenol A	ITGA5	Homo sapiens	[bisphenol A co-treated with Fulvestrant] results in increased methylation of ITGA5 gene	31601247
bisphenol A	ITGA5	Homo sapiens	bisphenol A results in decreased expression of ITGA5 mRNA	31715268|32981897
bisphenol A	ITGA5	Homo sapiens	bisphenol A results in decreased expression of ITGA5 protein	31675489|32981897
bisphenol A	ITGA5	Homo sapiens	[INS protein co-treated with Dexamethasone co-treated with 1-Methyl-3-isobutylxanthine co-treated with Indomethacin co-treated with bisphenol A] results in decreased expression of ITGA5 mRNA	28628672
bisphenol B	ITGA5	Homo sapiens	bisphenol B results in increased expression of ITGA5 protein	34186270
bisphenol F	ITGA5	Homo sapiens	bisphenol F results in increased expression of ITGA5 protein	34186270
Cadmium	ITGA5	Homo sapiens	[Cadmium Chloride results in increased abundance of Cadmium] which results in decreased expression of ITGA5 mRNA	29741670
Cadmium	ITGA5	Homo sapiens	Cadmium results in decreased expression of ITGA5 mRNA	20570719
Cadmium	ITGA5	Homo sapiens	Cadmium results in increased expression of ITGA5 mRNA	20570719
Cadmium Chloride	ITGA5	Homo sapiens	Cadmium Chloride results in decreased expression of ITGA5 mRNA	26472689
Cadmium Chloride	ITGA5	Homo sapiens	[Cadmium Chloride results in increased abundance of Cadmium] which results in decreased expression of ITGA5 mRNA	29741670
Cadmium Chloride	ITGA5	Homo sapiens	Cadmium Chloride results in increased expression of ITGA5 protein	28527916
carbendazim	ITGA5	Homo sapiens	carbendazim results in decreased expression of ITGA5 mRNA	25530041
Clioquinol	ITGA5	Homo sapiens	ITGA5 protein promotes the reaction [[Clioquinol binds to Copper] which results in increased phosphorylation of EGFR protein]	18346929
cobaltous chloride	ITGA5	Homo sapiens	cobaltous chloride results in increased expression of ITGA5 protein	16798617
Copper	ITGA5	Homo sapiens	[Chelating Agents binds to Copper] which results in increased expression of ITGA5 mRNA	30911355
Copper	ITGA5	Homo sapiens	ITGA5 protein promotes the reaction [[Clioquinol binds to Copper] which results in increased phosphorylation of EGFR protein]	18346929
Cosmetics	ITGA5	Homo sapiens	[Plasticizers co-treated with Cosmetics co-treated with Flame Retardants co-treated with perfluorooctanoic acid co-treated with Phytoestrogens] results in decreased expression of ITGA5 mRNA	33325755
DDT	ITGA5	Homo sapiens	DDT results in increased expression of ITGA5 mRNA	22902829
decabromobiphenyl ether	ITGA5	Homo sapiens	decabromobiphenyl ether results in decreased expression of ITGA5 protein	31675489
diallyl trisulfide	ITGA5	Homo sapiens	diallyl trisulfide results in decreased expression of ITGA5 protein	28741790
Dibutyl Phthalate	ITGA5	Homo sapiens	Dibutyl Phthalate results in increased expression of ITGA5 mRNA	34902519
Endosulfan	ITGA5	Homo sapiens	Endosulfan results in increased expression of ITGA5 mRNA	22902829
erucylphospho-N,N,N-trimethylpropylammonium	ITGA5	Homo sapiens	erucylphospho-N,N,N-trimethylpropylammonium results in increased expression of ITGA5 mRNA	29464035
Ethanol	ITGA5	Homo sapiens	Ethanol results in increased expression of ITGA5 mRNA	12720008
Flame Retardants	ITGA5	Homo sapiens	[Plasticizers co-treated with Cosmetics co-treated with Flame Retardants co-treated with perfluorooctanoic acid co-treated with Phytoestrogens] results in decreased expression of ITGA5 mRNA	33325755
glyphosate	ITGA5	Homo sapiens	glyphosate results in decreased expression of ITGA5 mRNA	31295307
Heptachlor	ITGA5	Homo sapiens	Heptachlor results in increased expression of ITGA5 mRNA	22902829
hexabrominated diphenyl ether 153	ITGA5	Homo sapiens	hexabrominated diphenyl ether 153 results in decreased expression of ITGA5 protein	31675489
Hydrogen Peroxide	ITGA5	Homo sapiens	Hydrogen Peroxide results in decreased expression of ITGA5 mRNA	12419474
Magnesium	ITGA5	Homo sapiens	[Aluminum Oxide co-treated with Magnesium] results in increased expression of ITGA5 protein	12209937
Ozone	ITGA5	Homo sapiens	[Aripiprazole co-treated with Ozone] results in increased expression of ITGA5 mRNA	31476115
Ozone	ITGA5	Homo sapiens	Ozone results in increased expression of ITGA5 mRNA	31476115
peracetylated N-azidoacetylmannosamine	ITGA5	Homo sapiens	peracetylated N-azidoacetylmannosamine results in decreased expression of ITGA5 mRNA	30181604
perfluoro-n-nonanoic acid	ITGA5	Homo sapiens	perfluoro-n-nonanoic acid results in increased expression of ITGA5 mRNA	32588087
perfluorooctanoic acid	ITGA5	Homo sapiens	[Plasticizers co-treated with Cosmetics co-treated with Flame Retardants co-treated with perfluorooctanoic acid co-treated with Phytoestrogens] results in decreased expression of ITGA5 mRNA	33325755
Plant Extracts	ITGA5	Homo sapiens	[Plant Extracts results in increased abundance of Cannabinoids] inhibits the reaction [TNF protein results in increased expression of ITGA5 mRNA]	31250491
Plasticizers	ITGA5	Homo sapiens	[Plasticizers co-treated with Cosmetics co-treated with Flame Retardants co-treated with perfluorooctanoic acid co-treated with Phytoestrogens] results in decreased expression of ITGA5 mRNA	33325755
potassium chromate(VI)	ITGA5	Homo sapiens	potassium chromate(VI) results in decreased expression of ITGA5 mRNA	22714537
quinoline	ITGA5	Homo sapiens	quinoline analog binds to and results in decreased activity of [ITGA5 protein binds to ITGB3 protein]	16984141
Silicon Dioxide	ITGA5	Homo sapiens	Silicon Dioxide analog results in increased expression of ITGA5 mRNA	25895662
Smoke	ITGA5	Homo sapiens	Smoke results in increased expression of ITGA5 mRNA	34520756
sodium arsenate	ITGA5	Homo sapiens	[sodium arsenate results in increased abundance of Arsenic] which results in increased expression of ITGA5 mRNA	32525701
sodium arsenite	ITGA5	Homo sapiens	sodium arsenite affects the methylation of ITGA5 gene	28589171
Sodium Selenite	ITGA5	Homo sapiens	Sodium Selenite results in increased expression of ITGA5 mRNA	18175754
tablysin-15, Tabanus yao	ITGA5	Homo sapiens	tablysin-15, Tabanus yao inhibits the reaction [VTN protein binds to [ITGA5 protein binds to ITGB3 protein]]	21475772
tert-Butylhydroperoxide	ITGA5	Homo sapiens	tert-Butylhydroperoxide results in decreased expression of ITGA5 mRNA	12419474
Tetrachlorodibenzodioxin	ITGA5	Homo sapiens	[Tetrachlorodibenzodioxin co-treated with 2-methyl-2H-pyrazole-3-carboxylic acid (2-methyl-4-o-tolylazophenyl)amide] results in decreased expression of ITGA5 mRNA	29704546
Tetrachlorodibenzodioxin	ITGA5	Homo sapiens	Tetrachlorodibenzodioxin results in increased expression of ITGA5 mRNA	16051281|22902829
Tetradecanoylphorbol Acetate	ITGA5	Homo sapiens	Atrazine inhibits the reaction [Tetradecanoylphorbol Acetate results in increased expression of ITGA5 mRNA]	24211529
Tetradecanoylphorbol Acetate	ITGA5	Homo sapiens	Tetradecanoylphorbol Acetate results in increased expression of ITGA5 mRNA	24211529
titanium dioxide	ITGA5	Homo sapiens	[Vitallium analog binds to titanium dioxide] which results in increased expression of ITGA5 mRNA	23825117
Tobacco Smoke Pollution	ITGA5	Homo sapiens	Tobacco Smoke Pollution affects the expression of ITGA5 protein	30291989
Tobacco Smoke Pollution	ITGA5	Homo sapiens	Tobacco Smoke Pollution results in increased expression of ITGA5 mRNA	27865774|33660061
tris(2-butoxyethyl) phosphate	ITGA5	Homo sapiens	tris(2-butoxyethyl) phosphate affects the expression of ITGA5 mRNA	29024780
Urethane	ITGA5	Homo sapiens	Urethane results in increased expression of ITGA5 mRNA	28818685
Vanadates	ITGA5	Homo sapiens	Vanadates results in increased expression of ITGA5 mRNA	22714537
Vitallium	ITGA5	Homo sapiens	[Vitallium analog binds to titanium dioxide] which results in increased expression of ITGA5 mRNA	23825117
4-(4-((5-(4,5-dimethyl-2-nitrophenyl)-2-furanyl)methylene)-4,5-dihydro-3-methyl-5-oxo-1H-pyrazol-1-yl)benzoic acid	LILRB1	Homo sapiens	4-(4-((5-(4,5-dimethyl-2-nitrophenyl)-2-furanyl)methylene)-4,5-dihydro-3-methyl-5-oxo-1H-pyrazol-1-yl)benzoic acid results in increased expression of LILRB1 mRNA	26191083
Aflatoxin B1	LILRB1	Homo sapiens	Aflatoxin B1 affects the expression of LILRB1 protein	20106945
Aflatoxin B1	LILRB1	Homo sapiens	Aflatoxin B1 results in decreased expression of LILRB1 mRNA	21632981
Aflatoxin B1	LILRB1	Homo sapiens	Aflatoxin B1 results in increased methylation of LILRB1 gene	27153756
Air Pollutants, Occupational	LILRB1	Homo sapiens	Air Pollutants, Occupational results in decreased expression of LILRB1 mRNA	23195993
Arsenic	LILRB1	Homo sapiens	Arsenic affects the methylation of LILRB1 gene	25304211
Arsenic Trioxide	LILRB1	Homo sapiens	Arsenic Trioxide results in decreased expression of LILRB1 mRNA	27829220
Arsenic Trioxide	LILRB1	Homo sapiens	Arsenic Trioxide results in increased expression of LILRB1 mRNA	22072212
Asbestos, Crocidolite	LILRB1	Homo sapiens	Asbestos, Crocidolite results in decreased expression of LILRB1 mRNA	29523930
Benzo(a)pyrene	LILRB1	Homo sapiens	Benzo(a)pyrene results in decreased expression of LILRB1 mRNA	21632981
Benzo(a)pyrene	LILRB1	Homo sapiens	Benzo(a)pyrene results in decreased methylation of LILRB1 5' UTR	27901495
benzo(e)pyrene	LILRB1	Homo sapiens	benzo(e)pyrene results in increased methylation of LILRB1 intron	30157460
cobaltous chloride	LILRB1	Homo sapiens	cobaltous chloride results in increased expression of LILRB1 mRNA	23052192
erucylphospho-N,N,N-trimethylpropylammonium	LILRB1	Homo sapiens	erucylphospho-N,N,N-trimethylpropylammonium results in increased expression of LILRB1 mRNA	29464035
Ethyl Methanesulfonate	LILRB1	Homo sapiens	Ethyl Methanesulfonate results in decreased expression of LILRB1 mRNA	23649840
Hydrogen Peroxide	LILRB1	Homo sapiens	Hydrogen Peroxide affects the expression of LILRB1 mRNA	21179406
Methyl Methanesulfonate	LILRB1	Homo sapiens	Methyl Methanesulfonate results in decreased expression of LILRB1 mRNA	23649840
Nickel	LILRB1	Homo sapiens	Nickel results in decreased expression of LILRB1 mRNA	23195993
Nickel	LILRB1	Homo sapiens	Nickel results in increased expression of LILRB1 mRNA	24768652|25583101
sodium bichromate	LILRB1	Homo sapiens	sodium bichromate results in decreased expression of LILRB1 mRNA	17685462
Tetrachlorodibenzodioxin	LILRB1	Homo sapiens	Tetrachlorodibenzodioxin results in decreased expression of LILRB1 mRNA	20106945|21632981
Zinc	LILRB1	Homo sapiens	Zinc deficiency results in increased expression of LILRB1 mRNA	22171008
2,2',4,4'-tetrabromodiphenyl ether	BGN	Homo sapiens	2,2',4,4'-tetrabromodiphenyl ether results in decreased expression of BGN protein	31675489
2,4,6-tribromophenol	BGN	Homo sapiens	2,4,6-tribromophenol results in decreased expression of BGN mRNA	31675489
4-(4-((5-(4,5-dimethyl-2-nitrophenyl)-2-furanyl)methylene)-4,5-dihydro-3-methyl-5-oxo-1H-pyrazol-1-yl)benzoic acid	BGN	Homo sapiens	4-(4-((5-(4,5-dimethyl-2-nitrophenyl)-2-furanyl)methylene)-4,5-dihydro-3-methyl-5-oxo-1H-pyrazol-1-yl)benzoic acid results in increased expression of BGN mRNA	26191083
4-(5-benzo(1,3)dioxol-5-yl-4-pyridin-2-yl-1H-imidazol-2-yl)benzamide	BGN	Homo sapiens	[NOG protein co-treated with Phenylmercuric Acetate co-treated with dorsomorphin co-treated with 4-(5-benzo(1,3)dioxol-5-yl-4-pyridin-2-yl-1H-imidazol-2-yl)benzamide] results in increased expression of BGN mRNA	27188386
4-(5-benzo(1,3)dioxol-5-yl-4-pyridin-2-yl-1H-imidazol-2-yl)benzamide	BGN	Homo sapiens	[NOG protein co-treated with trichostatin A co-treated with dorsomorphin co-treated with 4-(5-benzo(1,3)dioxol-5-yl-4-pyridin-2-yl-1H-imidazol-2-yl)benzamide] results in increased expression of BGN mRNA	27188386
4-(5-benzo(1,3)dioxol-5-yl-4-pyridin-2-yl-1H-imidazol-2-yl)benzamide	BGN	Homo sapiens	[NOG protein co-treated with Valproic Acid co-treated with dorsomorphin co-treated with 4-(5-benzo(1,3)dioxol-5-yl-4-pyridin-2-yl-1H-imidazol-2-yl)benzamide] results in increased expression of BGN mRNA	27188386
4-hydroxy-2-nonenal	BGN	Homo sapiens	[[BGN mRNA alternative form binds to OTUB1 protein] which binds to and results in decreased ubiquitination of and results in increased stability of SLC7A11 protein] which results in decreased abundance of 4-hydroxy-2-nonenal	35234341
Aflatoxin B1	BGN	Homo sapiens	Aflatoxin B1 results in decreased methylation of BGN intron	30157460
aflatoxin B2	BGN	Homo sapiens	aflatoxin B2 results in increased methylation of BGN intron	30157460
Aluminum Oxide	BGN	Homo sapiens	Aluminum Oxide results in increased expression of BGN mRNA	19464052
aristolochic acid I	BGN	Homo sapiens	aristolochic acid I results in decreased expression of BGN mRNA	33212167
Asbestos, Crocidolite	BGN	Homo sapiens	Asbestos, Crocidolite results in increased expression of BGN protein	29553831
Benzo(a)pyrene	BGN	Homo sapiens	Benzo(a)pyrene affects the methylation of BGN intron	30157460
Benzo(a)pyrene	BGN	Homo sapiens	Benzo(a)pyrene affects the methylation of BGN promoter	27901495
Benzo(a)pyrene	BGN	Homo sapiens	Benzo(a)pyrene results in increased methylation of BGN 5' UTR	27901495
benzo(e)pyrene	BGN	Homo sapiens	benzo(e)pyrene results in increased methylation of BGN intron	30157460
Cadmium	BGN	Homo sapiens	[Cadmium Chloride results in increased abundance of Cadmium] which results in decreased expression of BGN mRNA	29741670|35301059
Cadmium Chloride	BGN	Homo sapiens	[Cadmium Chloride results in increased abundance of Cadmium] which results in decreased expression of BGN mRNA	29741670|35301059
Chromium	BGN	Homo sapiens	Chromium results in decreased expression of BGN mRNA	21437242
cobaltous chloride	BGN	Homo sapiens	cobaltous chloride results in decreased secretion of BGN protein	22079246
Diazinon	BGN	Homo sapiens	Diazinon results in increased methylation of BGN gene	22964155
dorsomorphin	BGN	Homo sapiens	[NOG protein co-treated with Phenylmercuric Acetate co-treated with dorsomorphin co-treated with 4-(5-benzo(1,3)dioxol-5-yl-4-pyridin-2-yl-1H-imidazol-2-yl)benzamide] results in increased expression of BGN mRNA	27188386
dorsomorphin	BGN	Homo sapiens	[NOG protein co-treated with trichostatin A co-treated with dorsomorphin co-treated with 4-(5-benzo(1,3)dioxol-5-yl-4-pyridin-2-yl-1H-imidazol-2-yl)benzamide] results in increased expression of BGN mRNA	27188386
dorsomorphin	BGN	Homo sapiens	[NOG protein co-treated with Valproic Acid co-treated with dorsomorphin co-treated with 4-(5-benzo(1,3)dioxol-5-yl-4-pyridin-2-yl-1H-imidazol-2-yl)benzamide] results in increased expression of BGN mRNA	27188386
Ethanol	BGN	Homo sapiens	[[Gasoline co-treated with Ethanol] results in increased abundance of [Particulate Matter co-treated with Polycyclic Aromatic Hydrocarbons]] which results in decreased expression of BGN mRNA	29432896
Ferrous Compounds	BGN	Homo sapiens	[[BGN mRNA alternative form binds to OTUB1 protein] which binds to and results in decreased ubiquitination of and results in increased stability of SLC7A11 protein] which results in decreased abundance of Ferrous Compounds	35234341
Gasoline	BGN	Homo sapiens	[[Gasoline co-treated with Ethanol] results in increased abundance of [Particulate Matter co-treated with Polycyclic Aromatic Hydrocarbons]] which results in decreased expression of BGN mRNA	29432896
Lactic Acid	BGN	Homo sapiens	Lactic Acid affects the expression of BGN mRNA	30851411
Malondialdehyde	BGN	Homo sapiens	[[BGN mRNA alternative form binds to OTUB1 protein] which binds to and results in decreased ubiquitination of and results in increased stability of SLC7A11 protein] which results in decreased abundance of Malondialdehyde	35234341
Oxygen	BGN	Homo sapiens	Oxygen deficiency results in increased expression of BGN mRNA	26516004
Particulate Matter	BGN	Homo sapiens	[[Gasoline co-treated with Ethanol] results in increased abundance of [Particulate Matter co-treated with Polycyclic Aromatic Hydrocarbons]] which results in decreased expression of BGN mRNA	29432896
Phenylmercuric Acetate	BGN	Homo sapiens	[NOG protein co-treated with Phenylmercuric Acetate co-treated with dorsomorphin co-treated with 4-(5-benzo(1,3)dioxol-5-yl-4-pyridin-2-yl-1H-imidazol-2-yl)benzamide] results in increased expression of BGN mRNA	27188386
Phenylmercuric Acetate	BGN	Homo sapiens	Phenylmercuric Acetate results in increased expression of BGN mRNA	26272509
Plant Extracts	BGN	Homo sapiens	[Plant Extracts co-treated with Resveratrol] results in decreased expression of BGN mRNA	23557933
Polycyclic Aromatic Hydrocarbons	BGN	Homo sapiens	[[Gasoline co-treated with Ethanol] results in increased abundance of [Particulate Matter co-treated with Polycyclic Aromatic Hydrocarbons]] which results in decreased expression of BGN mRNA	29432896
potassium chromate(VI)	BGN	Homo sapiens	potassium chromate(VI) results in decreased expression of BGN mRNA	22714537
Tetrachlorodibenzodioxin	BGN	Homo sapiens	Tetrachlorodibenzodioxin affects the expression of BGN mRNA	22574217
Tobacco Smoke Pollution	BGN	Homo sapiens	Tobacco Smoke Pollution affects the expression of BGN protein	30291989
Tobacco Smoke Pollution	BGN	Homo sapiens	Tobacco Smoke Pollution results in decreased expression of BGN mRNA	33660061
tris(2-butoxyethyl) phosphate	BGN	Homo sapiens	tris(2-butoxyethyl) phosphate affects the expression of BGN mRNA	29024780
Uranium	BGN	Homo sapiens	Uranium affects the expression of BGN mRNA	15672453
uranyl acetate	BGN	Homo sapiens	uranyl acetate affects the expression of BGN mRNA	15672453

Moreover, we investigated the relationship between certain drugs and HMGSs through the CTD database (**Table 3**). Therefore, the administration of these drugs may exacerbate or alleviate the severity of UC. Further studies may be warranted to elucidate the underlying mechanisms to optimize drug choice and dosages, ultimately promoting better outcomes in UC management.

**Table 3:** The interaction between drug exposure and HMGSs

Chemical Name	Gene Symbol	Organism	Interaction	References PubMedID
Acetaminophen	GPR4	Homo sapiens	Acetaminophen results in decreased expression of GPR4 mRNA	22230336
Acetaminophen	GPR4	Homo sapiens	Acetaminophen results in increased expression of GPR4 mRNA	26690555
Anti-Inflammatory Agents	GPR4	Homo sapiens	Anti-Inflammatory Agents binds to and results in decreased activity of GPR4 protein	32370492
Anti-Inflammatory Agents	GPR4	Homo sapiens	[Anti-Inflammatory Agents binds to and results in decreased activity of GPR4 protein] inhibits the reaction [[Biological Factors binds to Sugars] which results in increased expression of IL1B mRNA]	32370492
Anti-Inflammatory Agents	GPR4	Homo sapiens	[Anti-Inflammatory Agents binds to and results in decreased activity of GPR4 protein] inhibits the reaction [[Biological Factors binds to Sugars] which results in increased expression of IL1B protein]	32370492
Anti-Inflammatory Agents	GPR4	Homo sapiens	[Anti-Inflammatory Agents binds to and results in decreased activity of GPR4 protein] inhibits the reaction [[Biological Factors binds to Sugars] which results in increased expression of IL6 mRNA]	32370492
Anti-Inflammatory Agents	GPR4	Homo sapiens	[Anti-Inflammatory Agents binds to and results in decreased activity of GPR4 protein] inhibits the reaction [[Biological Factors binds to Sugars] which results in increased expression of IL6 protein]	32370492
Anti-Inflammatory Agents	GPR4	Homo sapiens	[Anti-Inflammatory Agents binds to and results in decreased activity of GPR4 protein] inhibits the reaction [[Biological Factors binds to Sugars] which results in increased expression of MMP13 mRNA]	32370492
Anti-Inflammatory Agents	GPR4	Homo sapiens	[Anti-Inflammatory Agents binds to and results in decreased activity of GPR4 protein] inhibits the reaction [[Biological Factors binds to Sugars] which results in increased expression of MMP13 protein]	32370492
Anti-Inflammatory Agents	GPR4	Homo sapiens	[Anti-Inflammatory Agents binds to and results in decreased activity of GPR4 protein] inhibits the reaction [[Biological Factors binds to Sugars] which results in increased expression of MMP3 mRNA]	32370492
Anti-Inflammatory Agents	GPR4	Homo sapiens	[Anti-Inflammatory Agents binds to and results in decreased activity of GPR4 protein] inhibits the reaction [[Biological Factors binds to Sugars] which results in increased expression of MMP3 protein]	32370492
Anti-Inflammatory Agents	GPR4	Homo sapiens	[Anti-Inflammatory Agents binds to and results in decreased activity of GPR4 protein] inhibits the reaction [[Biological Factors binds to Sugars] which results in increased expression of NOS2 mRNA]	32370492
Anti-Inflammatory Agents	GPR4	Homo sapiens	[Anti-Inflammatory Agents binds to and results in decreased activity of GPR4 protein] inhibits the reaction [[Biological Factors binds to Sugars] which results in increased expression of NOS2 protein]	32370492
Anti-Inflammatory Agents	GPR4	Homo sapiens	[Anti-Inflammatory Agents binds to and results in decreased activity of GPR4 protein] inhibits the reaction [[Biological Factors binds to Sugars] which results in increased expression of PTGS2 mRNA]	32370492
Anti-Inflammatory Agents	GPR4	Homo sapiens	[Anti-Inflammatory Agents binds to and results in decreased activity of GPR4 protein] inhibits the reaction [[Biological Factors binds to Sugars] which results in increased expression of PTGS2 protein]	32370492
Anti-Inflammatory Agents	GPR4	Homo sapiens	[Anti-Inflammatory Agents binds to and results in decreased activity of GPR4 protein] inhibits the reaction [[Biological Factors binds to Sugars] which results in increased expression of TNF mRNA]	32370492
Anti-Inflammatory Agents	GPR4	Homo sapiens	[Anti-Inflammatory Agents binds to and results in decreased activity of GPR4 protein] inhibits the reaction [[Biological Factors binds to Sugars] which results in increased expression of TNF protein]	32370492
Antirheumatic Agents	GPR4	Homo sapiens	Antirheumatic Agents results in decreased expression of GPR4 mRNA	24449571
Biological Factors	GPR4	Homo sapiens	[Anti-Inflammatory Agents binds to and results in decreased activity of GPR4 protein] inhibits the reaction [[Biological Factors binds to Sugars] which results in increased expression of IL1B mRNA]	32370492
Biological Factors	GPR4	Homo sapiens	[Anti-Inflammatory Agents binds to and results in decreased activity of GPR4 protein] inhibits the reaction [[Biological Factors binds to Sugars] which results in increased expression of IL1B protein]	32370492
Biological Factors	GPR4	Homo sapiens	[Anti-Inflammatory Agents binds to and results in decreased activity of GPR4 protein] inhibits the reaction [[Biological Factors binds to Sugars] which results in increased expression of IL6 mRNA]	32370492
Biological Factors	GPR4	Homo sapiens	[Anti-Inflammatory Agents binds to and results in decreased activity of GPR4 protein] inhibits the reaction [[Biological Factors binds to Sugars] which results in increased expression of IL6 protein]	32370492
Biological Factors	GPR4	Homo sapiens	[Anti-Inflammatory Agents binds to and results in decreased activity of GPR4 protein] inhibits the reaction [[Biological Factors binds to Sugars] which results in increased expression of MMP13 mRNA]	32370492
Biological Factors	GPR4	Homo sapiens	[Anti-Inflammatory Agents binds to and results in decreased activity of GPR4 protein] inhibits the reaction [[Biological Factors binds to Sugars] which results in increased expression of MMP13 protein]	32370492
Biological Factors	GPR4	Homo sapiens	[Anti-Inflammatory Agents binds to and results in decreased activity of GPR4 protein] inhibits the reaction [[Biological Factors binds to Sugars] which results in increased expression of MMP3 mRNA]	32370492
Biological Factors	GPR4	Homo sapiens	[Anti-Inflammatory Agents binds to and results in decreased activity of GPR4 protein] inhibits the reaction [[Biological Factors binds to Sugars] which results in increased expression of MMP3 protein]	32370492
Biological Factors	GPR4	Homo sapiens	[Anti-Inflammatory Agents binds to and results in decreased activity of GPR4 protein] inhibits the reaction [[Biological Factors binds to Sugars] which results in increased expression of NOS2 mRNA]	32370492
Biological Factors	GPR4	Homo sapiens	[Anti-Inflammatory Agents binds to and results in decreased activity of GPR4 protein] inhibits the reaction [[Biological Factors binds to Sugars] which results in increased expression of NOS2 protein]	32370492
Biological Factors	GPR4	Homo sapiens	[Anti-Inflammatory Agents binds to and results in decreased activity of GPR4 protein] inhibits the reaction [[Biological Factors binds to Sugars] which results in increased expression of PTGS2 mRNA]	32370492
Biological Factors	GPR4	Homo sapiens	[Anti-Inflammatory Agents binds to and results in decreased activity of GPR4 protein] inhibits the reaction [[Biological Factors binds to Sugars] which results in increased expression of PTGS2 protein]	32370492
Biological Factors	GPR4	Homo sapiens	[Anti-Inflammatory Agents binds to and results in decreased activity of GPR4 protein] inhibits the reaction [[Biological Factors binds to Sugars] which results in increased expression of TNF mRNA]	32370492
Biological Factors	GPR4	Homo sapiens	[Anti-Inflammatory Agents binds to and results in decreased activity of GPR4 protein] inhibits the reaction [[Biological Factors binds to Sugars] which results in increased expression of TNF protein]	32370492
Biological Factors	GPR4	Homo sapiens	[Biological Factors binds to Sugars] which results in increased expression of GPR4 mRNA	32370492
Biological Factors	GPR4	Homo sapiens	[Biological Factors binds to Sugars] which results in increased expression of GPR4 protein	32370492
Estradiol	GPR4	Homo sapiens	[Estradiol co-treated with TGFB1 protein] results in increased expression of GPR4 mRNA	30165855
Lysophosphatidylcholines	GPR4	Homo sapiens	GPR4 protein promotes the reaction [Lysophosphatidylcholines results in increased expression of CASP3 protein]	34293432
Lysophosphatidylcholines	GPR4	Homo sapiens	GPR4 protein promotes the reaction [Lysophosphatidylcholines results in increased expression of IL18 protein]	34293432
Lysophosphatidylcholines	GPR4	Homo sapiens	GPR4 protein promotes the reaction [Lysophosphatidylcholines results in increased expression of IL1B protein]	34293432
Lysophosphatidylcholines	GPR4	Homo sapiens	GPR4 protein promotes the reaction [Lysophosphatidylcholines results in increased expression of IL33 protein]	34293432
Lysophosphatidylcholines	GPR4	Homo sapiens	GPR4 protein promotes the reaction [Lysophosphatidylcholines results in increased expression of NLRP3 protein]	34293432
Lysophosphatidylcholines	GPR4	Homo sapiens	Lysophosphatidylcholines results in increased expression of GPR4 mRNA	34293432
Lysophosphatidylcholines	GPR4	Homo sapiens	Lysophosphatidylcholines results in increased expression of GPR4 protein	34293432
Methotrexate	GPR4	Homo sapiens	Methotrexate results in decreased expression of GPR4 mRNA	24449571
quercitrin	GPR4	Homo sapiens	quercitrin results in increased expression of GPR4 mRNA	25193878
SCH772984	GPR4	Homo sapiens	SCH772984 inhibits the reaction [GPR4 protein affects the phosphorylation of MAPK1 protein]	33161135
SCH772984	GPR4	Homo sapiens	SCH772984 inhibits the reaction [GPR4 protein affects the phosphorylation of MAPK3 protein]	33161135
Valproic Acid	GPR4	Homo sapiens	Valproic Acid affects the expression of GPR4 mRNA	25979313
Valproic Acid	GPR4	Homo sapiens	Valproic Acid results in increased methylation of GPR4 gene	29154799
Acetaminophen	CYYR1	Homo sapiens	Acetaminophen results in increased expression of CYYR1 mRNA	29067470
bis(4-hydroxyphenyl)sulfone	CYYR1	Homo sapiens	bis(4-hydroxyphenyl)sulfone results in increased methylation of CYYR1 gene	31601247
Sunitinib	CYYR1	Homo sapiens	Sunitinib results in decreased expression of CYYR1 mRNA	31533062
Testosterone	CYYR1	Homo sapiens	Testosterone results in decreased expression of CYYR1 mRNA	33359661
Valproic Acid	CYYR1	Homo sapiens	Valproic Acid affects the expression of CYYR1 mRNA	25979313
Valproic Acid	CYYR1	Homo sapiens	Valproic Acid results in decreased expression of CYYR1 mRNA	23179753|25192806|28001369
abrine	ST3GAL2	Homo sapiens	abrine results in decreased expression of ST3GAL2 mRNA	31054353
Cannabidiol	ST3GAL2	Homo sapiens	Cannabidiol results in decreased expression of ST3GAL2 mRNA	33244087
Doxorubicin	ST3GAL2	Homo sapiens	Doxorubicin results in decreased expression of ST3GAL2 mRNA	29803840
Estradiol	ST3GAL2	Homo sapiens	Estradiol results in increased expression of ST3GAL2 mRNA	19429434
GSK-J4	ST3GAL2	Homo sapiens	GSK-J4 results in decreased expression of ST3GAL2 mRNA	29301935
Raloxifene Hydrochloride	ST3GAL2	Homo sapiens	Raloxifene Hydrochloride results in increased expression of ST3GAL2 mRNA	19429434
Sunitinib	ST3GAL2	Homo sapiens	Sunitinib results in decreased expression of ST3GAL2 mRNA	31533062
Tamoxifen	ST3GAL2	Homo sapiens	Tamoxifen results in increased expression of ST3GAL2 mRNA	19429434
Tretinoin	ST3GAL2	Homo sapiens	Tretinoin results in increased expression of ST3GAL2 mRNA	33167477
Valproic Acid	ST3GAL2	Homo sapiens	Valproic Acid affects the expression of ST3GAL2 mRNA	25979313
Ampicillin	CHST15	Homo sapiens	Ampicillin results in increased expression of CHST15 mRNA	21632981
Antirheumatic Agents	CHST15	Homo sapiens	Antirheumatic Agents results in decreased expression of CHST15 mRNA	24449571
belinostat	CHST15	Homo sapiens	belinostat results in increased expression of CHST15 mRNA	27188386
Biological Factors	CHST15	Homo sapiens	Biological Factors results in increased expression of CHST15 mRNA	32739440
Carbamazepine	CHST15	Homo sapiens	Carbamazepine affects the expression of CHST15 mRNA	25979313
CGP 52608	CHST15	Homo sapiens	CGP 52608 promotes the reaction [RORA protein binds to CHST15 gene]	28238834
Cyclosporine	CHST15	Homo sapiens	Cyclosporine results in decreased expression of CHST15 mRNA	25562108
Doxorubicin	CHST15	Homo sapiens	Doxorubicin results in decreased expression of CHST15 mRNA	29803840
entinostat	CHST15	Homo sapiens	entinostat results in increased expression of CHST15 mRNA	26272509
entinostat	CHST15	Homo sapiens	[NOG protein co-treated with entinostat co-treated with dorsomorphin co-treated with 4-(5-benzo(1,3)dioxol-5-yl-4-pyridin-2-yl-1H-imidazol-2-yl)benzamide] results in increased expression of CHST15 mRNA	27188386
epigallocatechin gallate	CHST15	Homo sapiens	[potassium chromate(VI) co-treated with epigallocatechin gallate] results in decreased expression of CHST15 mRNA	22079256
Estradiol	CHST15	Homo sapiens	[Estradiol co-treated with Progesterone] results in decreased expression of CHST15 mRNA	20660070
Estradiol	CHST15	Homo sapiens	[Estradiol co-treated with TGFB1 protein] results in increased expression of CHST15 mRNA	30165855
Fulvestrant	CHST15	Homo sapiens	[bisphenol A co-treated with Fulvestrant] results in increased methylation of CHST15 gene	31601247
ICG 001	CHST15	Homo sapiens	ICG 001 results in increased expression of CHST15 mRNA	26191083
Leflunomide	CHST15	Homo sapiens	Leflunomide results in increased expression of CHST15 mRNA	28988120
Lipopolysaccharides	CHST15	Homo sapiens	[S-(1,2-dichlorovinyl)cysteine co-treated with Lipopolysaccharides] results in decreased expression of CHST15 mRNA	35811015
Methotrexate	CHST15	Homo sapiens	Methotrexate results in decreased expression of CHST15 mRNA	24449571
Oxyquinoline	CHST15	Homo sapiens	Oxyquinoline results in increased expression of CHST15 mRNA	21632981
Panobinostat	CHST15	Homo sapiens	[NOG protein co-treated with Panobinostat co-treated with dorsomorphin co-treated with 4-(5-benzo(1,3)dioxol-5-yl-4-pyridin-2-yl-1H-imidazol-2-yl)benzamide] results in increased expression of CHST15 mRNA	27188386
Panobinostat	CHST15	Homo sapiens	Panobinostat results in increased expression of CHST15 mRNA	26272509
Progesterone	CHST15	Homo sapiens	[Estradiol co-treated with Progesterone] results in decreased expression of CHST15 mRNA	20660070
Quercetin	CHST15	Homo sapiens	Quercetin results in decreased expression of CHST15 mRNA	21632981
S-(1,2-dichlorovinyl)cysteine	CHST15	Homo sapiens	[S-(1,2-dichlorovinyl)cysteine co-treated with Lipopolysaccharides] results in decreased expression of CHST15 mRNA	35811015
sulforaphane	CHST15	Homo sapiens	sulforaphane results in increased expression of CHST15 mRNA	31838189
Thapsigargin	CHST15	Homo sapiens	Thapsigargin results in increased expression of CHST15 mRNA	22378314
Tretinoin	CHST15	Homo sapiens	Tretinoin results in decreased expression of CHST15 mRNA	23724009
Tretinoin	CHST15	Homo sapiens	Tretinoin results in increased expression of CHST15 mRNA	18052213|21934132|33167477
trichostatin A	CHST15	Homo sapiens	[NOG protein co-treated with trichostatin A co-treated with dorsomorphin co-treated with 4-(5-benzo(1,3)dioxol-5-yl-4-pyridin-2-yl-1H-imidazol-2-yl)benzamide] results in increased expression of CHST15 mRNA	27188386
trichostatin A	CHST15	Homo sapiens	trichostatin A results in increased expression of CHST15 mRNA	24935251|26272509
Valproic Acid	CHST15	Homo sapiens	[NOG protein co-treated with Valproic Acid co-treated with dorsomorphin co-treated with 4-(5-benzo(1,3)dioxol-5-yl-4-pyridin-2-yl-1H-imidazol-2-yl)benzamide] results in increased expression of CHST15 mRNA	27188386
Valproic Acid	CHST15	Homo sapiens	Valproic Acid affects the expression of CHST15 mRNA	25979313
Valproic Acid	CHST15	Homo sapiens	Valproic Acid results in increased expression of CHST15 mRNA	19101580|23179753|24383497|24935251|26272509|27188386|28001369
Vitamin K 3	CHST15	Homo sapiens	Vitamin K 3 affects the expression of CHST15 mRNA	23410634
Vorinostat	CHST15	Homo sapiens	Vorinostat results in increased expression of CHST15 mRNA	27188386
abrine	SLFN11	Homo sapiens	abrine results in decreased expression of SLFN11 mRNA	31054353
Calcitriol	SLFN11	Homo sapiens	Calcitriol results in increased expression of SLFN11 mRNA	16002434
Cytarabine	SLFN11	Homo sapiens	Cytarabine results in increased expression of SLFN11 mRNA	21198554
Dasatinib	SLFN11	Homo sapiens	Dasatinib results in decreased expression of SLFN11 mRNA	20579391
Disulfiram	SLFN11	Homo sapiens	[Disulfiram binds to Copper] which results in decreased expression of SLFN11 mRNA	24690739
Doxorubicin	SLFN11	Homo sapiens	Doxorubicin results in decreased expression of SLFN11 mRNA	29803840
entinostat	SLFN11	Homo sapiens	entinostat results in increased expression of SLFN11 mRNA	27188386
Enzyme Inhibitors	SLFN11	Homo sapiens	[Enzyme Inhibitors results in decreased activity of OGA protein] which results in increased O-linked glycosylation of SLFN11 protein	23301498
GSK-J4	SLFN11	Homo sapiens	GSK-J4 results in decreased expression of SLFN11 mRNA	29301935
incobotulinumtoxinA	SLFN11	Homo sapiens	incobotulinumtoxinA results in increased expression of SLFN11 mRNA	29522793
jinfukang	SLFN11	Homo sapiens	jinfukang results in increased expression of SLFN11 mRNA	27392435
Ribonucleotides	SLFN11	Homo sapiens	SLFN11 protein binds to Ribonucleotides	30528433
Temozolomide	SLFN11	Homo sapiens	Temozolomide results in increased expression of SLFN11 mRNA	31758290
trichostatin A	SLFN11	Homo sapiens	trichostatin A results in increased expression of SLFN11 mRNA	24935251
Valproic Acid	SLFN11	Homo sapiens	[NOG protein co-treated with Valproic Acid co-treated with dorsomorphin co-treated with 4-(5-benzo(1,3)dioxol-5-yl-4-pyridin-2-yl-1H-imidazol-2-yl)benzamide] results in increased expression of SLFN11 mRNA	27188386
Valproic Acid	SLFN11	Homo sapiens	Valproic Acid affects the expression of SLFN11 mRNA	25979313
Valproic Acid	SLFN11	Homo sapiens	Valproic Acid results in increased expression of SLFN11 mRNA	23179753|24383497|26272509|27188386|28001369
Vorinostat	SLFN11	Homo sapiens	Vorinostat results in increased expression of SLFN11 mRNA	27188386
abrine	GPR137B	Homo sapiens	abrine results in decreased expression of GPR137B mRNA	31054353
Antirheumatic Agents	GPR137B	Homo sapiens	Antirheumatic Agents results in decreased expression of GPR137B mRNA	24449571
Azathioprine	GPR137B	Homo sapiens	Azathioprine results in increased expression of GPR137B mRNA	22623647
Calcitriol	GPR137B	Homo sapiens	Calcitriol results in increased expression of GPR137B mRNA	21592394
Calcitriol	GPR137B	Homo sapiens	[Testosterone co-treated with Calcitriol] results in increased expression of GPR137B mRNA	21592394
CGP 52608	GPR137B	Homo sapiens	CGP 52608 promotes the reaction [RORA protein binds to GPR137B gene]	28238834
Coumestrol	GPR137B	Homo sapiens	Coumestrol results in decreased expression of GPR137B mRNA	19167446
Cyclophosphamide	GPR137B	Homo sapiens	Cyclophosphamide results in increased expression of GPR137B mRNA	21527772
Cyclosporine	GPR137B	Homo sapiens	Cyclosporine affects the expression of GPR137B mRNA	20106945
Cyclosporine	GPR137B	Homo sapiens	Cyclosporine results in increased expression of GPR137B mRNA	21632981|25562108
Dactinomycin	GPR137B	Homo sapiens	Dactinomycin results in increased expression of GPR137B mRNA	21527772
Demecolcine	GPR137B	Homo sapiens	Demecolcine results in increased expression of GPR137B mRNA	23649840
Doxorubicin	GPR137B	Homo sapiens	Doxorubicin results in decreased expression of GPR137B mRNA	29803840
Estradiol	GPR137B	Homo sapiens	Estradiol results in decreased expression of GPR137B mRNA	31614463
gardiquimod	GPR137B	Homo sapiens	gardiquimod results in increased expression of GPR137B mRNA	28003376
gardiquimod	GPR137B	Homo sapiens	Protein Kinase Inhibitors inhibits the reaction [gardiquimod results in increased expression of GPR137B mRNA]	28003376
GSK-J4	GPR137B	Homo sapiens	GSK-J4 results in increased expression of GPR137B mRNA	29301935
ICG 001	GPR137B	Homo sapiens	ICG 001 results in increased expression of GPR137B mRNA	26191083
Methapyrilene	GPR137B	Homo sapiens	Methapyrilene results in increased methylation of GPR137B intron	30157460
Protein Kinase Inhibitors	GPR137B	Homo sapiens	Protein Kinase Inhibitors inhibits the reaction [gardiquimod results in increased expression of GPR137B mRNA]	28003376
quercitrin	GPR137B	Homo sapiens	quercitrin results in increased expression of GPR137B mRNA	25193878
Temozolomide	GPR137B	Homo sapiens	Temozolomide results in increased expression of GPR137B mRNA	31758290
Testosterone	GPR137B	Homo sapiens	[Testosterone co-treated with Calcitriol] results in increased expression of GPR137B mRNA	21592394
Testosterone	GPR137B	Homo sapiens	Testosterone results in increased expression of GPR137B mRNA	21592394|33359661
Thapsigargin	GPR137B	Homo sapiens	Thapsigargin results in decreased expression of GPR137B mRNA	29453283
Tretinoin	GPR137B	Homo sapiens	Tretinoin results in increased expression of GPR137B mRNA	16249480|33167477
trichostatin A	GPR137B	Homo sapiens	trichostatin A results in increased expression of GPR137B mRNA	24935251
Tunicamycin	GPR137B	Homo sapiens	Tunicamycin results in decreased expression of GPR137B mRNA	29453283
Valproic Acid	GPR137B	Homo sapiens	Valproic Acid affects the expression of GPR137B mRNA	25979313
Valproic Acid	GPR137B	Homo sapiens	Valproic Acid results in increased expression of GPR137B mRNA	23179753|24935251|27188386|29154799
Vincristine	GPR137B	Homo sapiens	Vincristine results in increased expression of GPR137B mRNA	23649840
2-methyl-2H-pyrazole-3-carboxylic acid (2-methyl-4-o-tolylazophenyl)amide	ITGA5	Homo sapiens	[Tetrachlorodibenzodioxin co-treated with 2-methyl-2H-pyrazole-3-carboxylic acid (2-methyl-4-o-tolylazophenyl)amide] results in decreased expression of ITGA5 mRNA	29704546
abrine	ITGA5	Homo sapiens	abrine results in increased expression of ITGA5 mRNA	31054353
Acetone	ITGA5	Homo sapiens	Acetone results in increased expression of ITGA5 mRNA	12720008
AL-10 compound	ITGA5	Homo sapiens	AL-10 compound results in decreased metabolism of ITGA5 protein	20112294
arginyl-glycyl-aspartic acid	ITGA5	Homo sapiens	arginyl-glycyl-aspartic acid inhibits the reaction [Resveratrol binds to [ITGA5 protein binds to ITGB3 protein]]	16790523
Aripiprazole	ITGA5	Homo sapiens	[Aripiprazole co-treated with Ozone] results in increased expression of ITGA5 mRNA	31476115
Aspirin	ITGA5	Homo sapiens	Aspirin results in decreased expression of ITGA5 mRNA	15928584
beta-Naphthoflavone	ITGA5	Homo sapiens	beta-Naphthoflavone results in decreased expression of ITGA5 mRNA	32858204
bis(4-hydroxyphenyl)sulfone	ITGA5	Homo sapiens	bis(4-hydroxyphenyl)sulfone results in increased expression of ITGA5 protein	34186270
bis(4-hydroxyphenyl)sulfone	ITGA5	Homo sapiens	[INS protein co-treated with Dexamethasone co-treated with 1-Methyl-3-isobutylxanthine co-treated with Indomethacin co-treated with bis(4-hydroxyphenyl)sulfone] results in decreased expression of ITGA5 mRNA	28628672
bleomycetin	ITGA5	Homo sapiens	bleomycetin results in increased expression of ITGA5 mRNA	21040473
Bromodeoxyuridine	ITGA5	Homo sapiens	Bromodeoxyuridine results in increased expression of ITGA5 mRNA	7519154
Bromodeoxyuridine	ITGA5	Homo sapiens	Bromodeoxyuridine results in increased expression of ITGA5 protein	7519154
Cannabidiol	ITGA5	Homo sapiens	Cannabidiol inhibits the reaction [TNF protein results in increased expression of ITGA5 mRNA]	31250491
Cannabinoids	ITGA5	Homo sapiens	[Plant Extracts results in increased abundance of Cannabinoids] inhibits the reaction [TNF protein results in increased expression of ITGA5 mRNA]	31250491
CGP 52608	ITGA5	Homo sapiens	CGP 52608 promotes the reaction [RORA protein binds to ITGA5 gene]	28238834
Chelating Agents	ITGA5	Homo sapiens	[Chelating Agents binds to Copper] which results in increased expression of ITGA5 mRNA	30911355
Cisplatin	ITGA5	Homo sapiens	Cisplatin affects the expression of ITGA5 mRNA	23300844
Decitabine	ITGA5	Homo sapiens	Decitabine affects the expression of ITGA5 mRNA	23300844
Dexamethasone	ITGA5	Homo sapiens	[INS protein co-treated with Dexamethasone co-treated with 1-Methyl-3-isobutylxanthine co-treated with Indomethacin co-treated with bis(4-hydroxyphenyl)sulfone] results in decreased expression of ITGA5 mRNA	28628672
Dexamethasone	ITGA5	Homo sapiens	[INS protein co-treated with Dexamethasone co-treated with 1-Methyl-3-isobutylxanthine co-treated with Indomethacin co-treated with bisphenol A] results in decreased expression of ITGA5 mRNA	28628672
Diazepam	ITGA5	Homo sapiens	Diazepam results in increased expression of ITGA5 mRNA	19114084
Doxorubicin	ITGA5	Homo sapiens	Doxorubicin results in decreased expression of ITGA5 mRNA	29803840
Estradiol	ITGA5	Homo sapiens	[Estradiol co-treated with TGFB1 protein] results in increased expression of ITGA5 mRNA	30165855
Estradiol	ITGA5	Homo sapiens	[Progesterone co-treated with Estradiol] results in increased expression of ITGA5 mRNA	20226447
Folic Acid	ITGA5	Homo sapiens	Folic Acid affects the expression of ITGA5 mRNA	16361273
Fulvestrant	ITGA5	Homo sapiens	[bisphenol A co-treated with Fulvestrant] results in increased methylation of ITGA5 gene	31601247
Glucose	ITGA5	Homo sapiens	Glucose results in decreased expression of ITGA5 mRNA	31655124
GSK1210151A	ITGA5	Homo sapiens	GSK1210151A inhibits the reaction [TGFB1 protein results in increased expression of ITGA5 mRNA]	26644586
GSK1210151A	ITGA5	Homo sapiens	GSK1210151A results in decreased expression of ITGA5 mRNA	26644586
Hydrocortisone	ITGA5	Homo sapiens	Hydrocortisone results in increased expression of ITGA5 mRNA	21267416
ICG 001	ITGA5	Homo sapiens	ICG 001 results in increased expression of ITGA5 mRNA	26191083
Indomethacin	ITGA5	Homo sapiens	[INS protein co-treated with Dexamethasone co-treated with 1-Methyl-3-isobutylxanthine co-treated with Indomethacin co-treated with bis(4-hydroxyphenyl)sulfone] results in decreased expression of ITGA5 mRNA	28628672
Indomethacin	ITGA5	Homo sapiens	[INS protein co-treated with Dexamethasone co-treated with 1-Methyl-3-isobutylxanthine co-treated with Indomethacin co-treated with bisphenol A] results in decreased expression of ITGA5 mRNA	28628672
Ivermectin	ITGA5	Homo sapiens	Ivermectin results in decreased expression of ITGA5 protein	32959892
(+)-JQ1 compound	ITGA5	Homo sapiens	(+)-JQ1 compound inhibits the reaction [TGFB1 protein results in increased expression of ITGA5 mRNA]	26644586
(+)-JQ1 compound	ITGA5	Homo sapiens	(+)-JQ1 compound results in decreased expression of ITGA5 mRNA	26644586
Leuprolide	ITGA5	Homo sapiens	Leuprolide inhibits the reaction [IGF1 protein results in increased expression of and affects the localization of [ITGA5 protein binds to ITGB3 protein]]	17143537
Methapyrilene	ITGA5	Homo sapiens	Methapyrilene results in increased methylation of ITGA5 intron	30157460
Methotrexate	ITGA5	Homo sapiens	Methotrexate results in increased expression of ITGA5 mRNA	17400583
Phytoestrogens	ITGA5	Homo sapiens	[Plasticizers co-treated with Cosmetics co-treated with Flame Retardants co-treated with perfluorooctanoic acid co-treated with Phytoestrogens] results in decreased expression of ITGA5 mRNA	33325755
Progesterone	ITGA5	Homo sapiens	[Progesterone co-treated with Estradiol] results in increased expression of ITGA5 mRNA	20226447
Progesterone	ITGA5	Homo sapiens	Progesterone results in increased expression of ITGA5 mRNA	20226447|21795739
Quercetin	ITGA5	Homo sapiens	Quercetin results in increased expression of ITGA5 mRNA	30152185
Resveratrol	ITGA5	Homo sapiens	arginyl-glycyl-aspartic acid inhibits the reaction [Resveratrol binds to [ITGA5 protein binds to ITGB3 protein]]	16790523
Resveratrol	ITGA5	Homo sapiens	[ITGA5 protein binds to ITGB3 protein] promotes the reaction [Resveratrol results in increased phosphorylation of MAPK1 protein]	16790523
Resveratrol	ITGA5	Homo sapiens	[ITGA5 protein binds to ITGB3 protein] promotes the reaction [Resveratrol results in increased phosphorylation of MAPK3 protein]	16790523
Resveratrol	ITGA5	Homo sapiens	[ITGA5 protein binds to ITGB3 protein] promotes the reaction [Resveratrol results in increased phosphorylation of TP53 protein]	16790523
Resveratrol	ITGA5	Homo sapiens	Resveratrol binds to [ITGA5 protein binds to ITGB3 protein]	16790523
Resveratrol	ITGA5	Homo sapiens	Resveratrol results in decreased expression of ITGA5 protein	18089832
Rosiglitazone	ITGA5	Homo sapiens	Rosiglitazone results in increased expression of ITGA5 protein	19467017
Rosiglitazone	ITGA5	Homo sapiens	Tretinoin promotes the reaction [Rosiglitazone results in increased expression of ITGA5 protein]	19467017
Sunitinib	ITGA5	Homo sapiens	Sunitinib results in increased expression of ITGA5 mRNA	31533062
Temozolomide	ITGA5	Homo sapiens	Temozolomide results in decreased expression of ITGA5 mRNA	31758290
Tetracycline	ITGA5	Homo sapiens	Tetracycline results in increased expression of ITGA5 mRNA	28882639
Topotecan	ITGA5	Homo sapiens	ITGA5 protein affects the susceptibility to Topotecan	16217747
Tretinoin	ITGA5	Homo sapiens	Tretinoin promotes the reaction [Rosiglitazone results in increased expression of ITGA5 protein]	19467017
Troglitazone	ITGA5	Homo sapiens	Troglitazone results in increased expression of ITGA5 mRNA	19140230
Valproic Acid	ITGA5	Homo sapiens	CEBPA protein affects the reaction [Valproic Acid results in decreased expression of ITGA5 mRNA]	32623605
Valproic Acid	ITGA5	Homo sapiens	Valproic Acid results in decreased expression of ITGA5 mRNA	29154799|32623605
Antirheumatic Agents	LILRB1	Homo sapiens	Antirheumatic Agents results in decreased expression of LILRB1 mRNA	24449571
Catechin	LILRB1	Homo sapiens	[Catechin co-treated with Grape Seed Proanthocyanidins] results in decreased expression of LILRB1 mRNA	24763279
Grape Seed Proanthocyanidins	LILRB1	Homo sapiens	[Catechin co-treated with Grape Seed Proanthocyanidins] results in decreased expression of LILRB1 mRNA	24763279
Levonorgestrel	LILRB1	Homo sapiens	[testosterone undecanoate co-treated with Levonorgestrel] results in increased expression of LILRB1 mRNA	19074003
Lipopolysaccharides	LILRB1	Homo sapiens	[S-(1,2-dichlorovinyl)cysteine affects the susceptibility to Lipopolysaccharides] which results in increased expression of LILRB1 mRNA	35811015
Lipopolysaccharides	LILRB1	Homo sapiens	[S-(1,2-dichlorovinyl)cysteine co-treated with Lipopolysaccharides] results in decreased expression of LILRB1 mRNA	35811015
Methapyrilene	LILRB1	Homo sapiens	Methapyrilene results in increased methylation of LILRB1 intron	30157460
Methotrexate	LILRB1	Homo sapiens	Methotrexate results in decreased expression of LILRB1 mRNA	17400583
S-(1,2-dichlorovinyl)cysteine	LILRB1	Homo sapiens	[S-(1,2-dichlorovinyl)cysteine affects the susceptibility to Lipopolysaccharides] which results in increased expression of LILRB1 mRNA	35811015
S-(1,2-dichlorovinyl)cysteine	LILRB1	Homo sapiens	[S-(1,2-dichlorovinyl)cysteine co-treated with Lipopolysaccharides] results in decreased expression of LILRB1 mRNA	35811015
S-(1,2-dichlorovinyl)cysteine	LILRB1	Homo sapiens	S-(1,2-dichlorovinyl)cysteine results in decreased expression of LILRB1 mRNA	35811015
testosterone undecanoate	LILRB1	Homo sapiens	[testosterone undecanoate co-treated with Levonorgestrel] results in increased expression of LILRB1 mRNA	19074003
testosterone undecanoate	LILRB1	Homo sapiens	testosterone undecanoate results in increased expression of LILRB1 mRNA	19074003
Valproic Acid	LILRB1	Homo sapiens	Valproic Acid results in increased methylation of LILRB1 gene	29154799
Vincristine	LILRB1	Homo sapiens	Vincristine results in decreased expression of LILRB1 mRNA	23649840
Acetaminophen	BGN	Homo sapiens	Acetaminophen results in increased expression of BGN mRNA	22230336
Antineoplastic Agents, Immunological	BGN	Homo sapiens	[Antineoplastic Agents, Immunological results in decreased susceptibility to Antineoplastic Agents, Immunological] which results in increased expression of BGN mRNA alternative form	35234341
Antineoplastic Agents, Immunological	BGN	Homo sapiens	BGN mRNA alternative form results in decreased susceptibility to Antineoplastic Agents, Immunological	35234341
Antineoplastic Agents, Immunological	BGN	Homo sapiens	erastin inhibits the reaction [[Antineoplastic Agents, Immunological results in decreased susceptibility to Antineoplastic Agents, Immunological] which results in increased expression of BGN mRNA alternative form]	35234341
beta-Naphthoflavone	BGN	Homo sapiens	beta-Naphthoflavone results in decreased expression of BGN mRNA	32858204
Cisplatin	BGN	Homo sapiens	Cisplatin affects the expression of BGN mRNA	23300844
Dasatinib	BGN	Homo sapiens	Dasatinib results in increased expression of BGN mRNA	20579391
Decitabine	BGN	Homo sapiens	Decitabine affects the expression of BGN mRNA	23300844
Dexamethasone	BGN	Homo sapiens	Dexamethasone results in decreased expression of BGN mRNA	25047013
Diazepam	BGN	Homo sapiens	Diazepam results in increased expression of BGN mRNA	19114084
Doxorubicin	BGN	Homo sapiens	Doxorubicin affects the expression of BGN protein	29385562
Doxorubicin	BGN	Homo sapiens	Doxorubicin results in increased expression of BGN mRNA	29803840
erastin	BGN	Homo sapiens	BGN mRNA alternative form inhibits the reaction [erastin results in decreased expression of MKI67 protein]	35234341
erastin	BGN	Homo sapiens	erastin inhibits the reaction [[Antineoplastic Agents, Immunological results in decreased susceptibility to Antineoplastic Agents, Immunological] which results in increased expression of BGN mRNA alternative form]	35234341
erastin	BGN	Homo sapiens	erastin results in decreased expression of BGN mRNA alternative form	35234341
Estradiol	BGN	Homo sapiens	[Estradiol co-treated with TGFB1 protein] results in increased expression of BGN mRNA	30165855
Estradiol	BGN	Homo sapiens	Estradiol results in increased expression of BGN mRNA	19429434|21185374
Glutathione	BGN	Homo sapiens	[[BGN mRNA alternative form binds to OTUB1 protein] which binds to and results in decreased ubiquitination of and results in increased stability of SLC7A11 protein] which results in increased chemical synthesis of Glutathione	35234341
ICG 001	BGN	Homo sapiens	ICG 001 results in increased expression of BGN mRNA	26191083
Isotretinoin	BGN	Homo sapiens	Isotretinoin results in increased expression of BGN mRNA	20436886
Methapyrilene	BGN	Homo sapiens	Methapyrilene results in increased methylation of BGN intron	30157460
Paclitaxel	BGN	Homo sapiens	BGN protein affects the susceptibility to Paclitaxel	16217747
Raloxifene Hydrochloride	BGN	Homo sapiens	Raloxifene Hydrochloride results in increased expression of BGN mRNA	19429434
Resveratrol	BGN	Homo sapiens	[Plant Extracts co-treated with Resveratrol] results in decreased expression of BGN mRNA	23557933
Temozolomide	BGN	Homo sapiens	Temozolomide results in increased expression of BGN mRNA	31758290
trichostatin A	BGN	Homo sapiens	[NOG protein co-treated with trichostatin A co-treated with dorsomorphin co-treated with 4-(5-benzo(1,3)dioxol-5-yl-4-pyridin-2-yl-1H-imidazol-2-yl)benzamide] results in increased expression of BGN mRNA	27188386
trichostatin A	BGN	Homo sapiens	trichostatin A results in increased expression of BGN mRNA	24935251|26272509
Valproic Acid	BGN	Homo sapiens	[NOG protein co-treated with Valproic Acid co-treated with dorsomorphin co-treated with 4-(5-benzo(1,3)dioxol-5-yl-4-pyridin-2-yl-1H-imidazol-2-yl)benzamide] results in increased expression of BGN mRNA	27188386
Valproic Acid	BGN	Homo sapiens	Valproic Acid results in decreased expression of BGN mRNA	29154799
Valproic Acid	BGN	Homo sapiens	Valproic Acid results in increased expression of BGN mRNA	23179753|26272509
Valproic Acid	BGN	Homo sapiens	Valproic Acid results in increased methylation of BGN gene	29154799

## Discussion

As yet, the genetic abnormalities involved in the exacerbation of UC have not been adequately explored. The identification of these genetic abnormalities may have great clinical implications in targeting UC treatment and hold the promise for achieving clinical disease remission of UC.

Based on multiple bioinformatic methods, we identified 9 gene signatures (HMGSs) and one potential therapeutic small-molecule drug (Exisulind) of the exacerbation of UC. Verification in multiple datasets suggested that the 9 HMGSs exhibit good diagnostic capacity in predicting the severity of UC. Furthermore, the 9 HMGSs were also good biomakers of UC. Thus, our research here provided a resource for future studies and highlighted 9 potential therapeutic targets. In addition, we generated a novel genotyping scheme based on the 9 HMGSs and then found that UC patients in cluster C1 were susceptible to benefit from CS-IV treatment. A further GSEA enrichment analysis indicated that cluster C1 was indeed enriched in several energy metabolism-associated signaling pathways, including the oxidative phosphorylation, pentose and glucuronate interconversions and citrate cycle TCA cycle pathways. Corticosteroids plays an important role in regulating both energy metabolism and glucose homeostasis (35). The unique energy metabolism pattern of cluster C1 was most likely responsible for the sensitivity to corticosteroids therapy. Numerous studies have shown evidence supporting that cellular energy metabolism pathways are altered during the differentiation and activation of immune cells (36). In addition, metabolic products and intermediates also regulate the cellular function of several immune cells (37). Our study yielded similar result that cluster C1 had a remarkably distinct immune cell infiltration characterization compared to cluster C2. The cluster C2 had a significant higher level of CD4+ T cells. CD4+ T cells have been reported as a major initiators in the disease process of UC (38). Blockade and depletion of CD4+ T cells are an effective means of treatments for IBD (39). Therefore, a higher degree of CD4+ T cells in cluster C2 may contributed to the higher disease severity. Overall, our study provided a convenient and valuable tool to predict severity of UC and screen UC patients suitable for CS-IV treatment. Intravenous administration of corticosteroids can achieve therapeutic effects with reduced oral administration dosages, and can alleviate adverse reactions associated with oral corticosteroids such as gastrointestinal discomfort. Additionally, it is worth noting that most UC patients that receive corticosteroid therapy *via* the intravenous route have a higher degree of disease severity. It is worth noting that a majority of UC patients who receive corticosteroid administration *via* the intravenous route have a higher degree of disease severity. Thus, our molecular typing scheme may be specific only to the severe UC patient population in predicting therapeutic responsiveness.

Exisulind, or what is also termed “Sulindac sulfone”, is a metabolite of sulindac and is also a non-steroidal anti-inflammatory drug (NSAID). NSAIDs have generally been were considered to be related to an increased risk of mucosal ulceration. But a high-quality meta-analyze showed that NSAIDs did not elicit exacerbations and serious complications of the IBD (40). By the way, the anti-tumor application of Exisulind has already been explored in Phase I or Phase II clinical trials, suggesting that Exisulind is well tolerated with relatively few adverse effects (41–44). Although Exisulind has only weak anti-inflammatory effect, extensive experimental data have proved that Exisulind have a therapeutic potential to prevent and cure many diseases of the colon. The mTORC1 pathway has been reported to modulate the regulation and differentiation of immune cells, and then ameliorate colitis (45). It is worthy to mention that Exisulin has been shown to inhibit the mTORC1 pathway by directly targeting voltage-dependent anion channel 1 and 2 (46). Regulation of the mTORC1 pathway may be one of the underlying mechanisms responsible for the therapeutic effectiveness of Exisulind.

Additionally, our molecular docking results suggested that GPR4 is the protein with the highest docking score with Exisulind. Thus, GPR4 protein might be another potential targets of Exisulind in UC. As a pro-inflammatory G protein-coupled receptor (GPCR), GPR4 showed a higher expression level in vascular endothelial cells (47–49). GPR4 has a significant role in regulating endothelium-blood cell interaction and leukocyte infiltration. In addition, GPR4 exhibits capability to regulate vascular permeability and tissue edema under inflammatory conditions (50–52). Numerous experimental animal studies revealed that GPR4 is involved in the development and progression of UC. GPR4 played a protective pole in dextran sulfate sodium-induced acute colitis mouse model (53–55). Therefore, the inhibition of GPR4 could be a underlying mechanism responsible for the therapeutic effects of Exisulind on UC.

We present a comprehensive review aimed at investigating the effect of environmental toxins exposure on HMGSs expression levels - a phenomenon that may play a potential role in influencing the severity of UC. It is noteworthy to mention that this effect is not limited solely to environmental toxins as some drug exposure may trigger similar effects. Our objective is to shed light on the crucial interplay between external factors and HMGSs, and its clinical implications in the context of UC pathogenesis. Our research provides novel insights and resources that can facilitate a more comprehensive examination of the complex relationship between UC progression and environmental toxin exposure. Consequently, these findings can potentially inform novel perspectives for guiding clinical treatment strategies for UC patients, thereby improving the standard of care for this condition.

This study provided new ideas and materials for the personalized clinical treatment plans for patients with UC, although some limitations to the present study need to be considered. First of all, this research only included a bioinformatics analysis, lacking further experimental verification as a solid foundation. Secondly, one of the imitations of our study is that this research is a retrospective study rather than a prospectively trial. Our identification of potential therapeutic agents for UC was based on computational methods, thus necessitating further *in vitro* and *in vivo* experimental validation and exploration of underlying mechanisms. Therefore, future follow-up studies with prospective clinical trials and mechanistic exploration are required for corroboration of our findings.

## Conclusion

In summary, we explored the genetic abnormalities involved in the exacerbation of UC based on microarray technology. By combining WGCNA and random forest algorithm, we identified 9 gene signatures (HMGSs) of the exacerbation of UC. Then a novel genotyping scheme was generated based on the 9 HMGSs, dividing patients into two subtypes (cluster C1 and cluster C2). Patients in cluster C1 were susceptible to benefit from CS-IV treatment. Subsequently, we identified a small molecule drug (Exisulind) with potential therapeutic effects for UC. We also provided a comprehensive review of the environmental toxins and drug exposures that potentially impact the progression of UC. Thus, our research contributed to the development of personalized clinical management and treatment regimens for UC.

## Data availability statement

Publicly available datasets were analyzed in this study. This data can be found in Gene Expression Omnibus (GEO) database (https://www.ncbi.nlm.nih.gov/geo/). The accession numbers can be found in the article/**Supplementary Material**.

## Ethics statement

The studies involving human subjects were evaluated and authorized by the Ethics Committees of the Jiangsu Province Hospital. Participants in the study provided written, informed consent to participate in this study. Consent was obtained in writing from individuals for the use of any potentially discernible data or images in this article.

## Author contributions

Conceptualization, YW; Formal analysis, YW and R-HZ; Methodology, HZ and YW; Project administration, Z-NF; Supervision, H-YW; Writing - original draft, YW; Writing - review and editing, Z-NF and HW; Sample collection and PCR, X-HJ. All authors have contributed to this article and have approved the final version submitted.

## Supplementary material

Supplementary Figure 1 Flowchart illustrating the workflow of this study.

Supplementary Figure 2 Flow chart of the WGCNA analysis pipeline.

Supplementary Figure 3 Determination of soft-threshold power in the WGCNA in GSE109142 cohort. **(A)** Analysis of the scale-free index for various soft-threshold powers (β). **(B)** Analysis of the mean connectivity for various soft-threshold powers. **(C)** Clustering dendrogram of UC patients in the GSE109142 cohort. Identification of modules closely associated with Mayo score. **(D)** Dendrogram of all differentially expressed genes clustered based on the measurement of dissimilarity (1-TOM). The color band shows the results obtained from the automatic single-block analysis. **(E)** Number of genes in each module.

Supplementary Figure 4 Determination of soft-threshold power in the WGCNA in GSE92415 cohort. **(A)** Analysis of the scale-free index for various soft-threshold powers (β). **(B)** Analysis of the mean connectivity for various soft-threshold powers. **(C)** Clustering dendrogram of UC patients in the GSE92415 cohort. Identification of modules closely associated with Mayo score. **(D)** Dendrogram of all differentially expressed genes clustered based on the measurement of dissimilarity (1-TOM). The color band shows the results obtained from the automatic single-block analysis. **(E)** Number of genes in each module.

Supplementary Figure 5 PCA,UMAP and tSNE cluster representation of the 9 HMGSs expression patterns. ROC curve of first principal component, second principal component and the sum of first and second principal component for differentiating UC from normal samples.

Supplementary Figure 6
**(A)** Correlations between Mayo score and HMGSs assessed by Spearman correlation in GSE92415 cohort. **(B)** Based on upper and lower quartiles of the set of Mayo scores in GSE92415 cohort, UC patients were stratified to high- (red), moderate- (blue), and low- (green) Mayo score groups. Boxplots showing the expression levels of the 9 HMGSs across different Mayo score group. **(C)** Boxplots showing the expression levels of HMGSs in UC intestinal samples (red) and normal intestinal samples (blue).

Supplementary Figure 7 ROC curve illustrating the potential of HMGSs to differentiate between UC patients with high- and low- Mayo scores in GSE92415 cohort.

Supplementary Figure 8 Independent dataset (GSE73661) verification results. **(A)** Boxplots showing the expression levels of the HMGSs in the UC patient groups with different Mayo endoscopic scores. **(B)** Boxplots showing the expression levels of HMGSs in UC intestinal samples (red) and normal intestinal samples (blue). **(C)** ROC curve illustrating the potential of HMGSs to differentiate between UC patients with high- and low- Mayo endoscopic scores in GSE73661 cohort.

Supplementary Figure 9 GSEA enrichment analysis results in GSE109142 cohort. The top five most enriched KEGG pathways in cluster C1 **(A)** and Cluster C2 **(B)** were shown. **(C)** Box-plot highlighting the differences in immune cell infiltration between the cluster C1 (red) and Cluster C2 (blue).

Supplementary Figure 10 Experimental verification of gene expression levels of GPR4, ST3GAL2, and LILRB1 using qRT-PCR.

Supplementary Table 5 The immune cell infiltration of each UC patient of GSE109142 cohort evaluated by ImmuCellAI database.
