# Corrigendum: Unveiling the key genes, environmental toxins, and drug exposures in modulating the severity of ulcerative colitis: a comprehensive analysis

**DOI:** 10.3389/fimmu.2023.1323997

**Published:** 2023-11-14

**Authors:** Yao Wang, Hao Zhuang, Xiao-han Jiang, Rui-han Zou, Hai-yang Wang, Zhi-ning Fan

**Affiliations:** Digestive Endoscopy Department, Jiangsu Province Hospital, The First Affiliated Hospital with Nanjing Medical University, Nanjing, China

**Keywords:** ulcerative colitis, microarray, biomarker, genomics, bioinformatics

In the published article, there was an error in the Funding statement. We did not introduce or acknowledge the funding source(s) for this research in the article. The correct Funding statement appears below.

