# Supplementary figures and images for: Unveiling the key genes, environmental toxins, and drug exposures in modulating the severity of ulcerative colitis: a comprehensive analysis
Source: Front Immunol. 2023 Jul 19;14:1162458. doi: 10.3389/fimmu.2023.1162458 (PMC10394652; doi:10.3389/fimmu.2023.1162458)

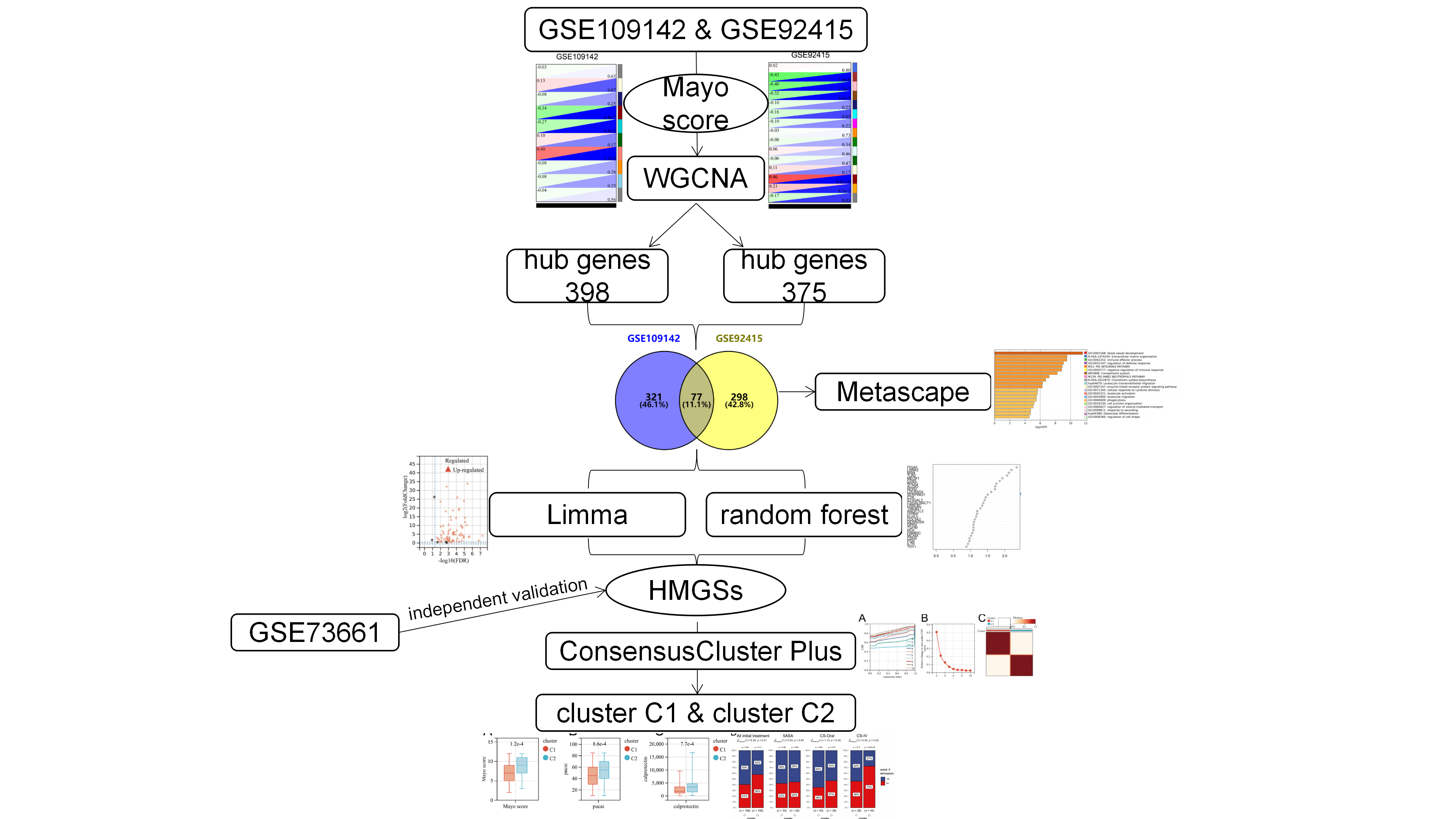

Supplement: Supplementary Figure 1 — Flowchart illustrating the workflow of this study. [file Image_1.png]

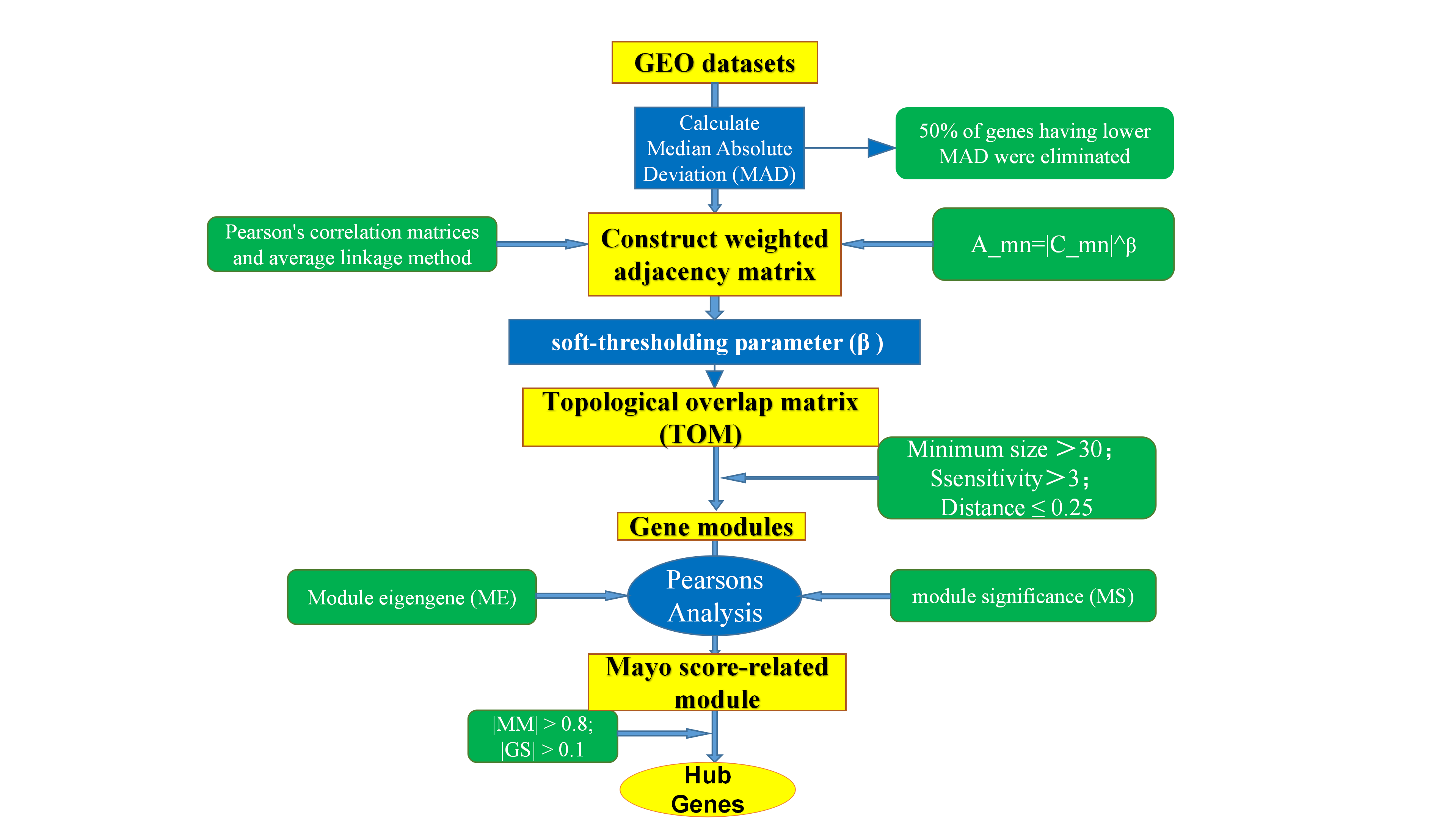

Supplement: Supplementary Figure 2 — Flow chart of the WGCNA analysis pipeline. [file Image_2.png]

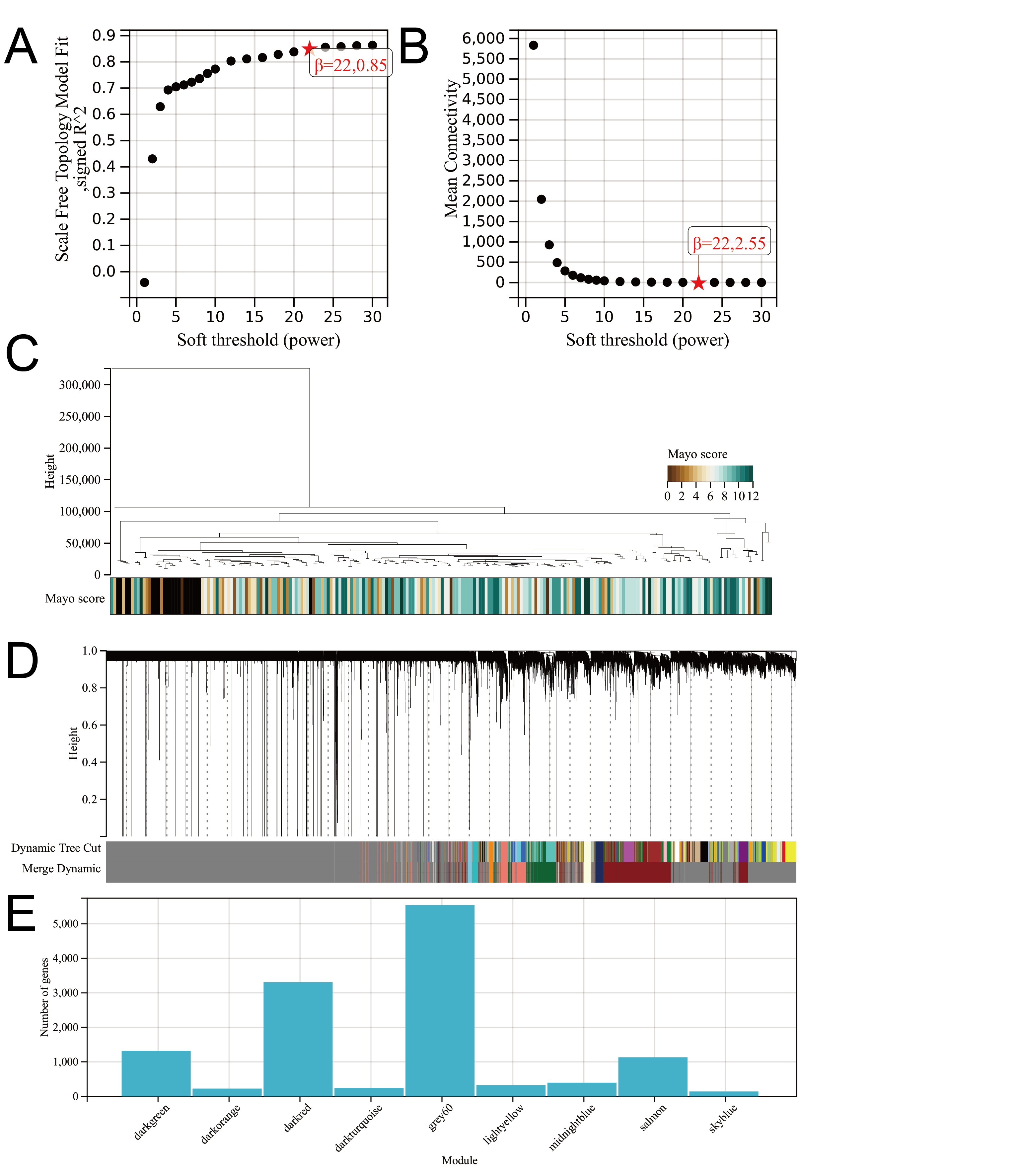

Supplement: Supplementary Figure 3 — Determination of soft-threshold power in the WGCNA in GSE109142 cohort. (A) Analysis of the scale-free index for various soft-threshold powers (β). (B) Analysis of the mean connectivity for various soft-threshold powers. (C) Clustering dendrogram of UC patients in the GSE109142 cohort. Identification of modules closely associated with Mayo score. (D) Dendrogram of all differentially expressed genes clustered based on the measurement of dissimilarity (1-TOM). The color band shows the results obtained from the automatic single-block analysis. (E) Number of genes in each module. [file Image_3.png]

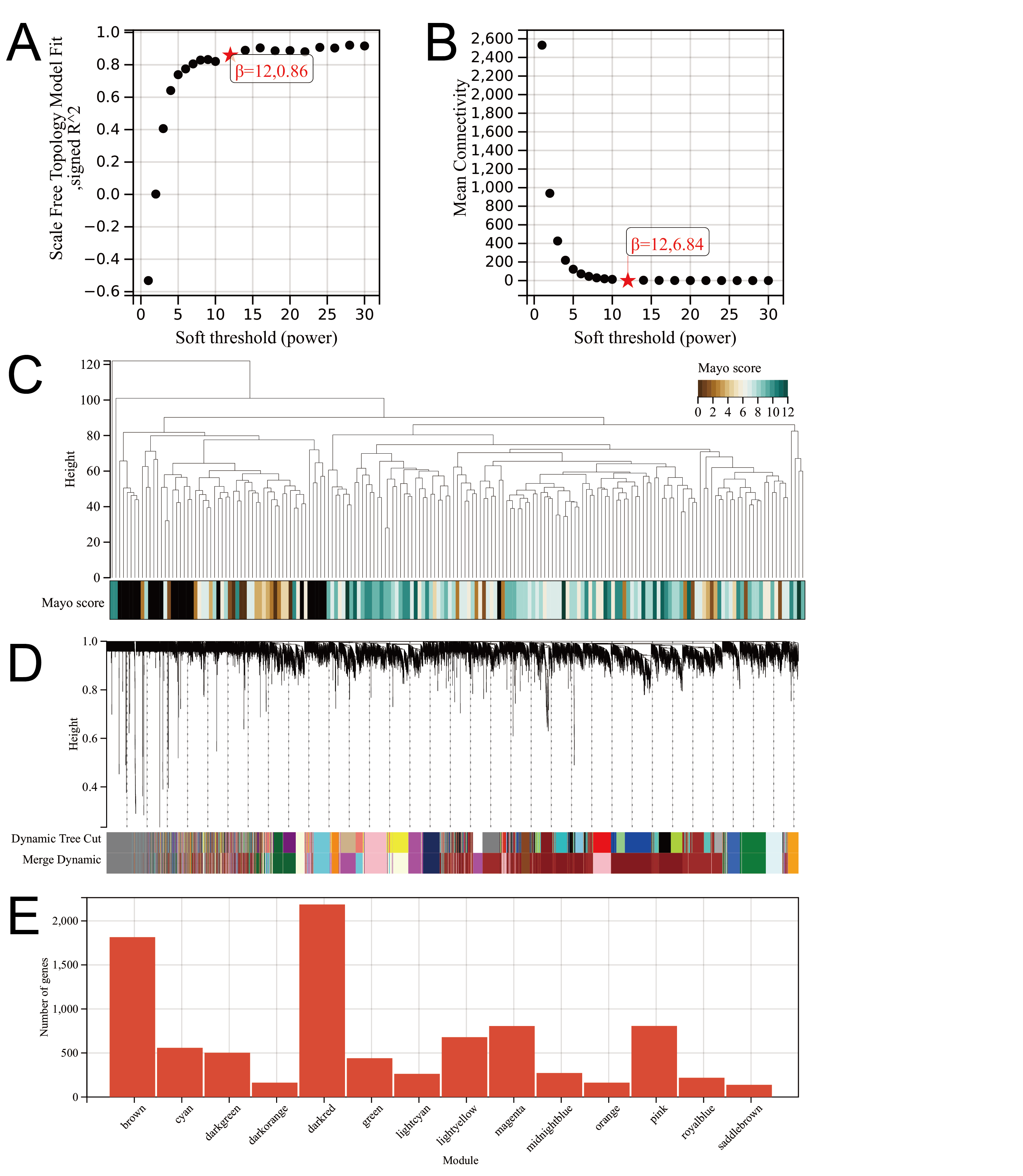

Supplement: Supplementary Figure 4 — Determination of soft-threshold power in the WGCNA in GSE92415 cohort. (A) Analysis of the scale-free index for various soft-threshold powers (β). (B) Analysis of the mean connectivity for various soft-threshold powers. (C) Clustering dendrogram of UC patients in the GSE92415 cohort. Identification of modules closely associated with Mayo score. (D) Dendrogram of all differentially expressed genes clustered based on the measurement of dissimilarity (1-TOM). The color band shows the results obtained from the automatic single-block analysis. (E) Number of genes in each module. [file Image_4.png]

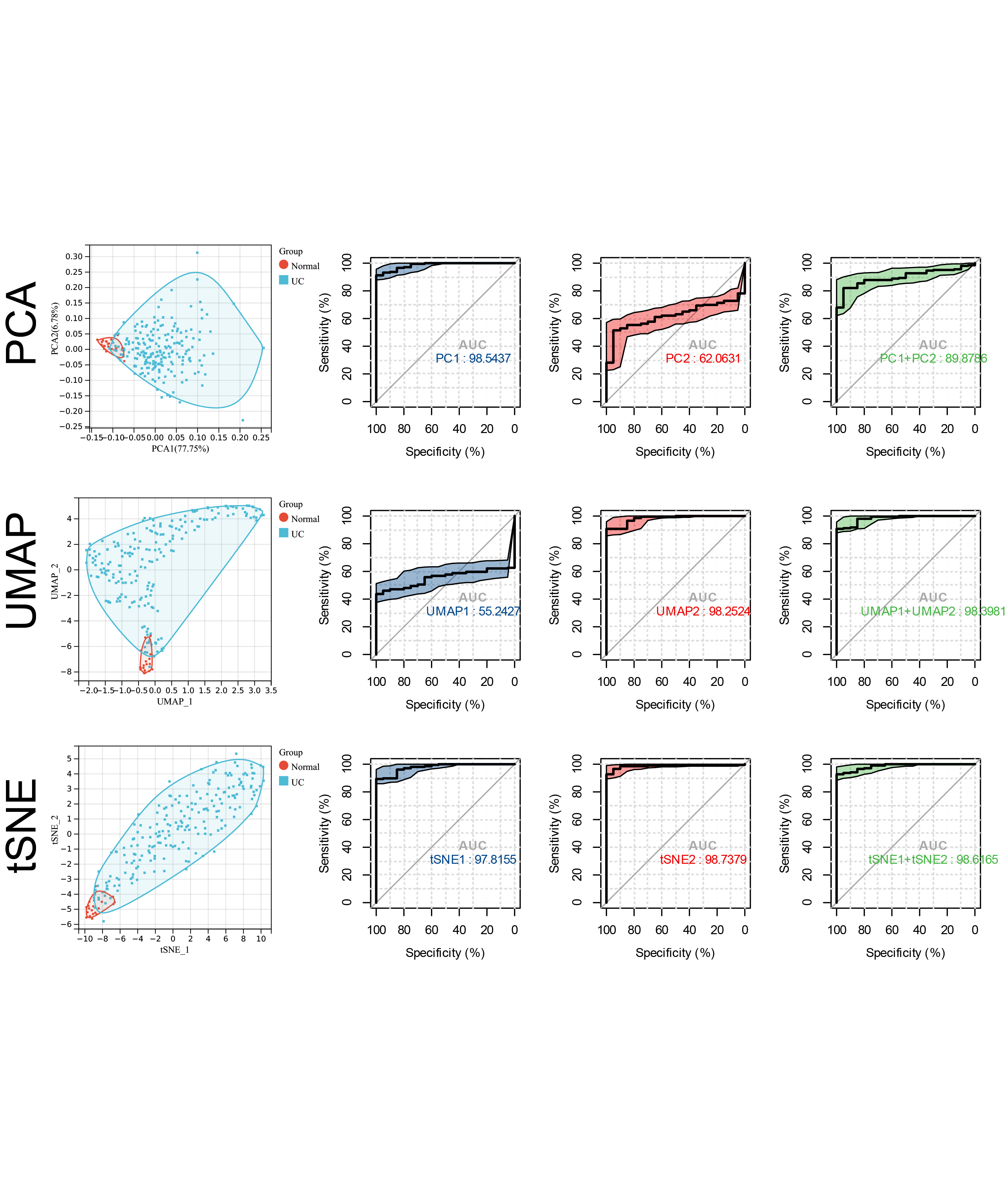

Supplement: Supplementary Figure 5 — PCA,UMAP and tSNE cluster representation of the 9 HMGSs expression patterns. ROC curve of first principal component, second principal component and the sum of first and second principal component for differentiating UC from normal samples. [file Image_5.png]

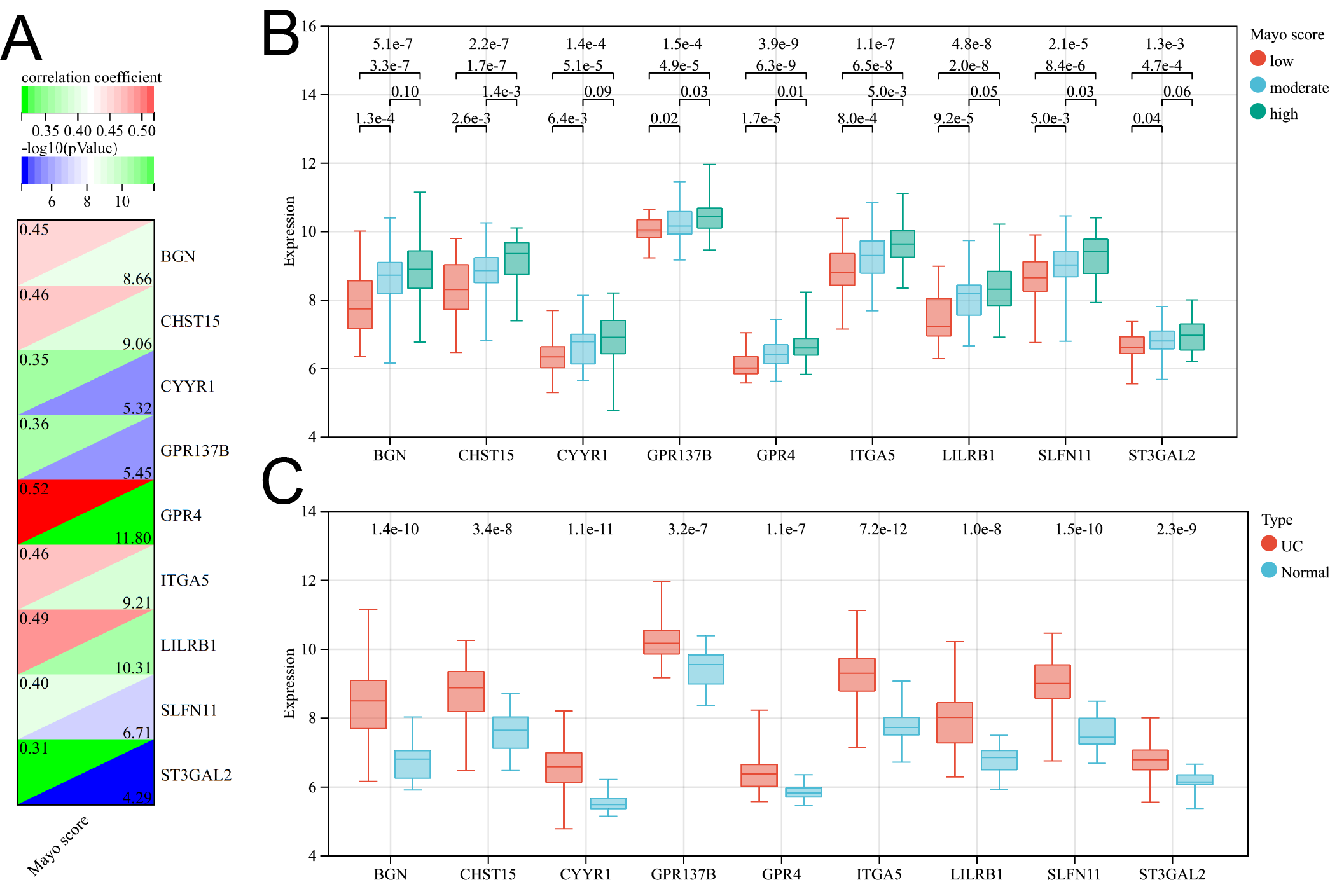

Supplement: Supplementary Figure 6 — (A) Correlations between Mayo score and HMGSs assessed by Spearman correlation in GSE92415 cohort. (B) Based on upper and lower quartiles of the set of Mayo scores in GSE92415 cohort, UC patients were stratified to high- (red), moderate- (blue), and low- (green) Mayo score groups. Boxplots showing the expression levels of the 9 HMGSs across different Mayo score group. (C) Boxplots showing the expression levels of HMGSs in UC intestinal samples (red) and normal intestinal samples (blue). [file Image_6.png]

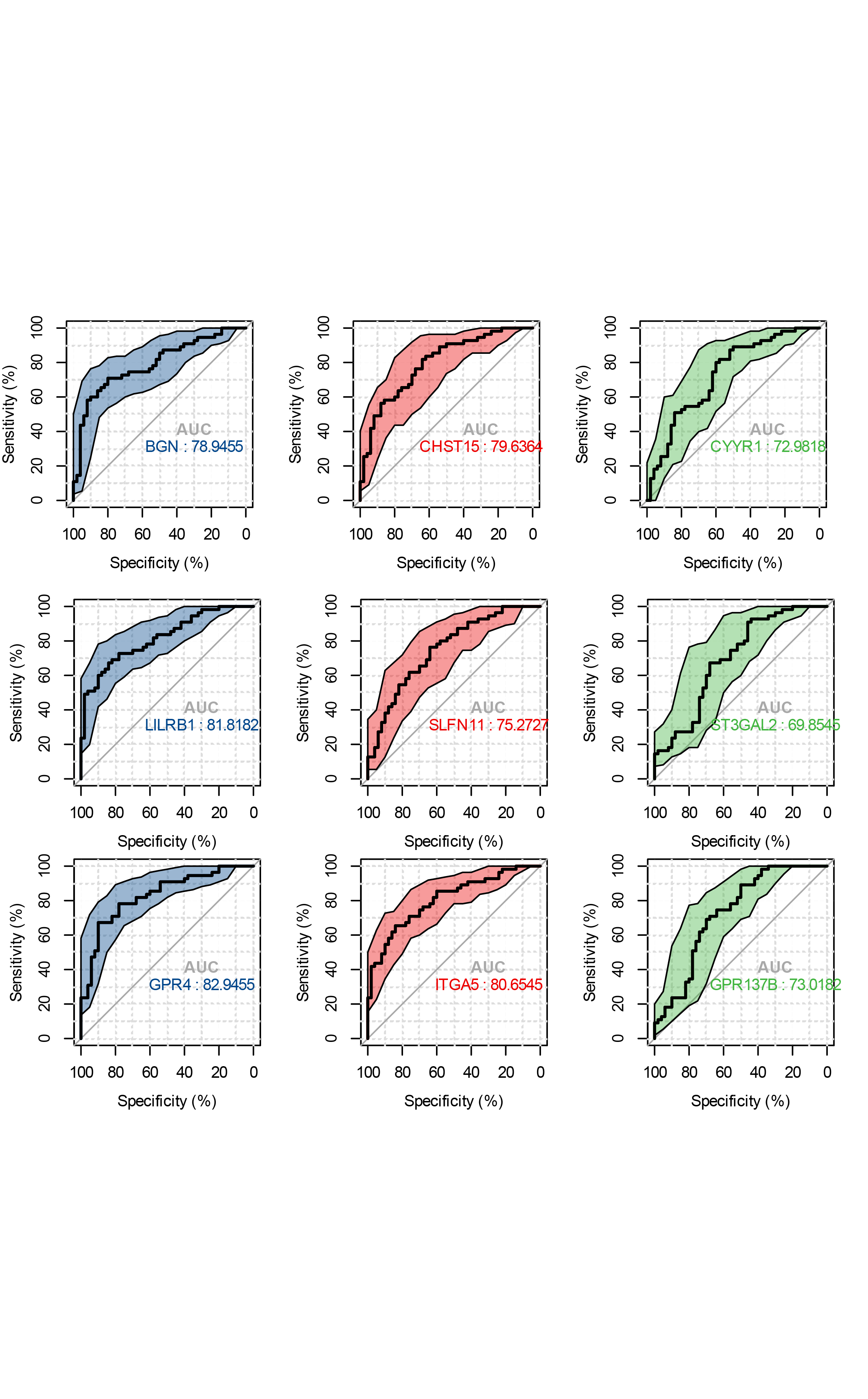

Supplement: Supplementary Figure 7 — ROC curve illustrating the potential of HMGSs to differentiate between UC patients with high- and low- Mayo scores in GSE92415 cohort. [file Image_7.png]

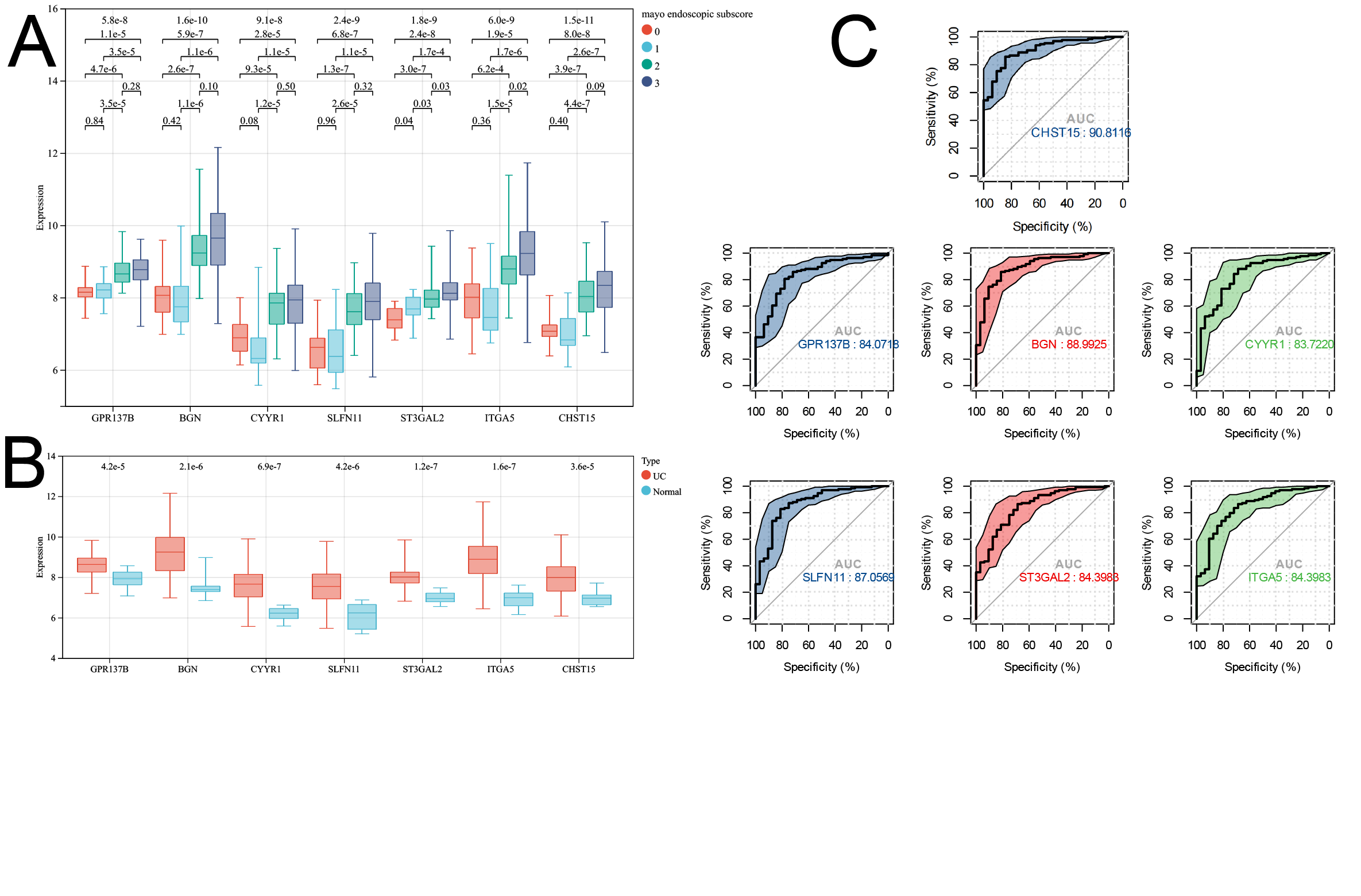

Supplement: Supplementary Figure 8 — Independent dataset (GSE73661) verification results. (A) Boxplots showing the expression levels of the HMGSs in the UC patient groups with different Mayo endoscopic scores. (B) Boxplots showing the expression levels of HMGSs in UC intestinal samples (red) and normal intestinal samples (blue). (C) ROC curve illustrating the potential of HMGSs to differentiate between UC patients with high- and low- Mayo endoscopic scores in GSE73661 cohort. [file Image_8.png]

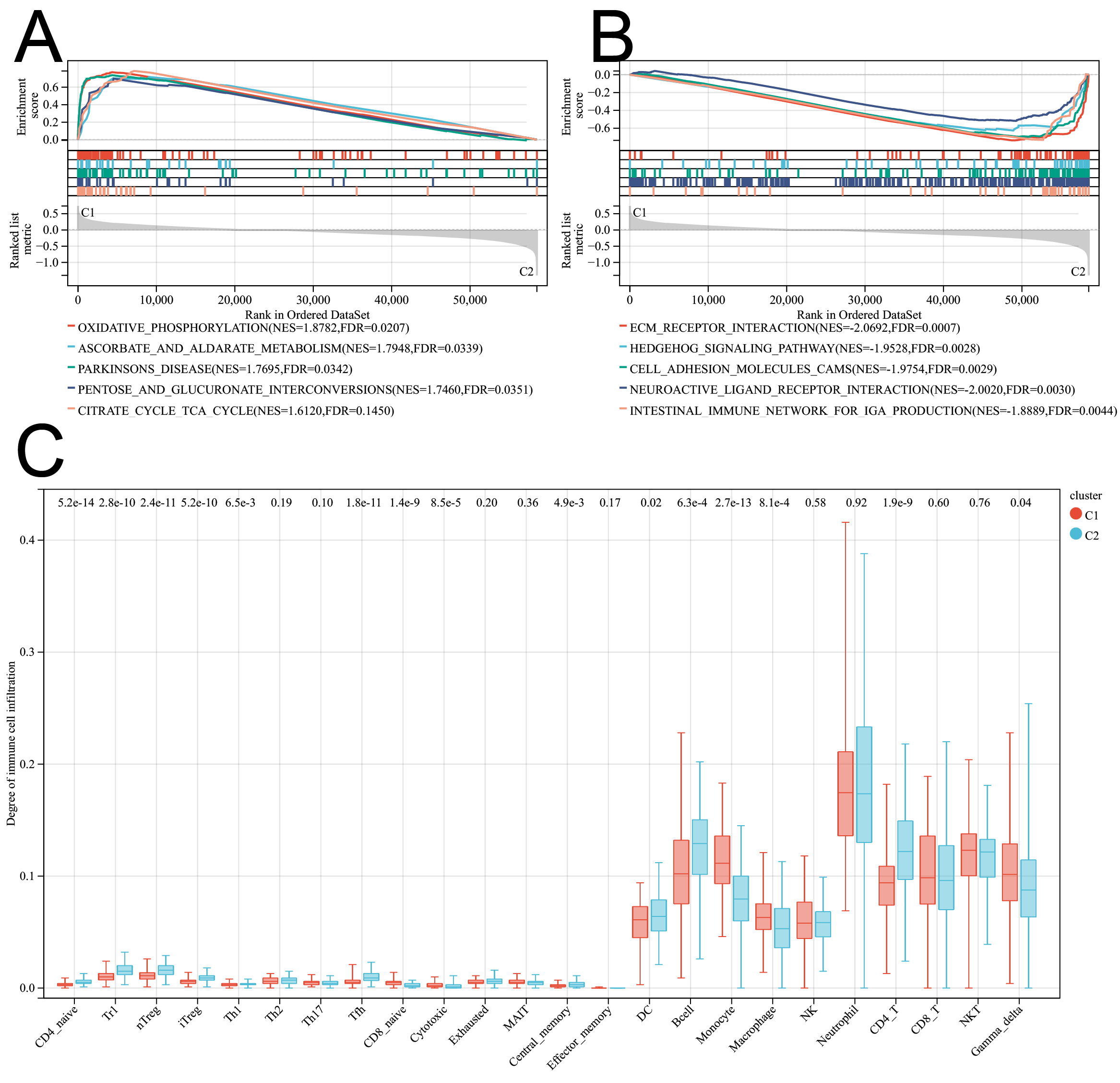

Supplement: Supplementary Figure 9 — GSEA enrichment analysis results in GSE109142 cohort. The top five most enriched KEGG pathways in cluster C1 (A) and Cluster C2 (B) were shown. (C) Box-plot highlighting the differences in immune cell infiltration between the cluster C1 (red) and Cluster C2 (blue). [file Image_9.png]

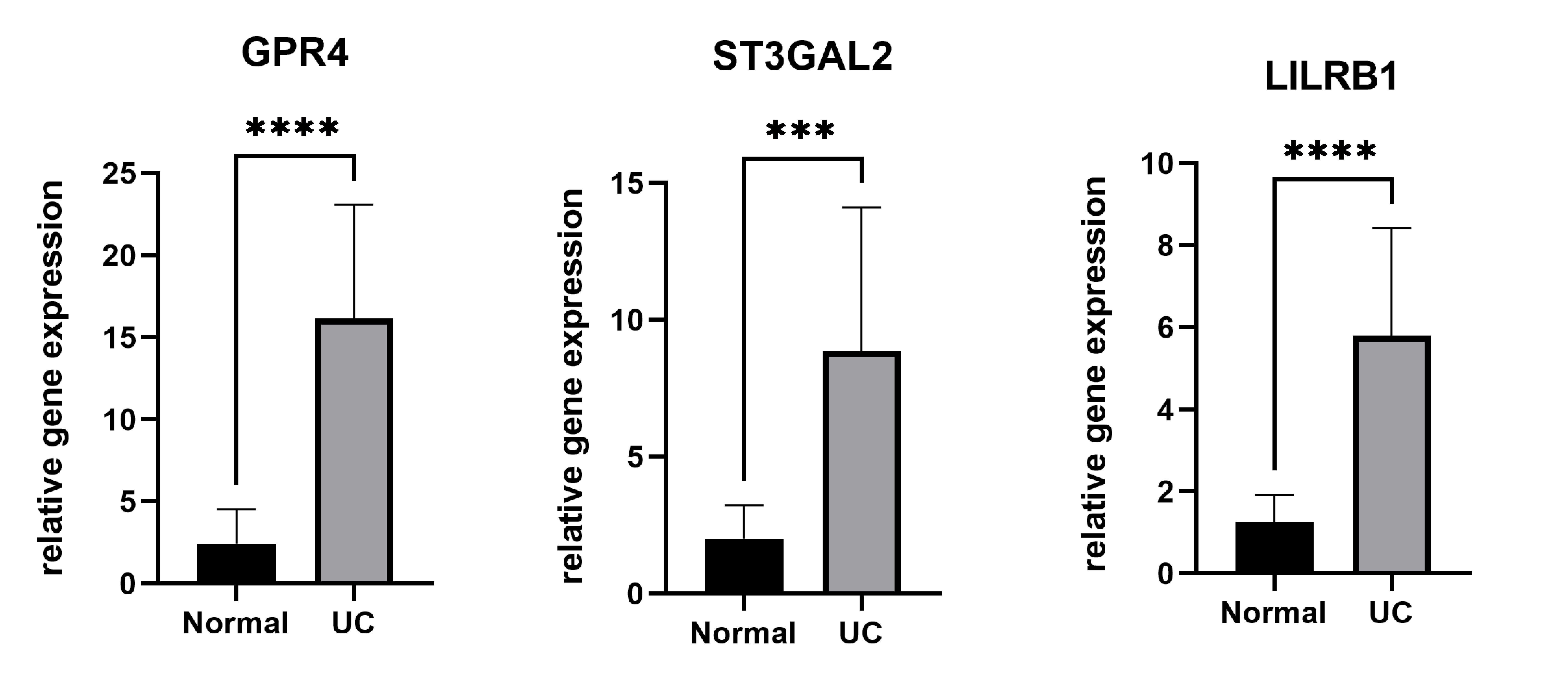

Supplement: Supplementary Figure 10 — Experimental verification of gene expression levels of GPR4, ST3GAL2, and LILRB1 using qRT-PCR. [file Image_10.jpeg]
